# Therapeutic Evaluation of Alginate from Brown Seaweeds: A Comparative Study of *Turbinaria ornata* and *Hormophysa cuneiformis*

**DOI:** 10.3390/ph18111720

**Published:** 2025-11-12

**Authors:** Mostafa M. El-Sheekh, Eman Bases, Shimaa M. El Shafay, Rania A. El-Shenody, Mostafa E. Elshobary, Abdel Hady A. Abdel Wahab, Wesam E. Yousuf, Dorya I. Essa, Samar Sami Alkafaas

**Affiliations:** 1 Botany and Microbiology Department, Faculty of Science, Tanta University, Tanta 31527, Egypt; mostafaelsheikh@science.tanta.edu.eg (M.M.E.-S.); eman.bases@science.tanta.edu.eg (E.B.); shimaa.elshafay@science.tanta.edu.eg (S.M.E.S.); rania.abdelsamad@science.tanta.edu.eg (R.A.E.-S.); dorya_eesa@science.tanta.edu.eg (D.I.E.); 2 Aquaculture Research, Alfred Wegener Institute (AWI)–Helmholtz Centre for Polar and Marine Research, Am Handelshafen, 27570 Bremerhaven, Germany; 3 Department of Cancer Biology, National Cancer Institute, Cairo University, Cairo 11796, Egypt; abdelhady.abdelwahab@nci.cu.edu.eg; 4 Biochemistry Division, Chemistry Department, Faculty of Science, Tanta University, Tanta 31527, Egypt; wessam-pg147370@scienc.tanta.edu.eg; 5 Molecular Cell Biology Unit, Division of Biochemistry, Department of Chemistry, Faculty of Science, Tanta University, Tanta 31527, Egypt; samar.alkafas@science.tanta.edu.eg

**Keywords:** polysaccharide, antioxidant, anti-inflammatory, antidiabetic, neuroprotective, hepatoprotective, molecular docking, COX-1 inhibition

## Abstract

**Background:** Alginate is a naturally occurring anionic polysaccharide extracted from brown marine algae and widely explored for biomedical applications due to its biocompatibility and functional versatility. This study aims to extract and compare alginates from two Red Sea brown algae, *Turbinaria ornata* (TA) and *Hormophysa cuneiformis* (HA), and to evaluate how structural differences influence their therapeutic properties. **Methods:** Alginate was isolated by sequential acid–alkaline extraction and characterized using FTIR, XRD, TGA, elemental analysis, and HPLC. Biological activities were assessed through antioxidant, anti-inflammatory, antidiabetic, neuroprotective, and hepatoprotective assays, supported by molecular docking and gene ontology interaction analysis. **Results:** Distinct physicochemical variations were observed between HA and TA. TA exhibited stronger antioxidant (IC_50_ = 25.89 µg/mL), anti-inflammatory (COX-1 IC_50_ = 69.61 µg/mL), antidiabetic (α-amylase IC_50_ = 45.14 µg/mL), and hepatoprotective activities (IC_50_ = 118.21 µg/mL), whereas HA displayed superior neuroprotective potential through butyrylcholinesterase inhibition (IC_50_ = 39.01 µg/mL). Molecular docking supported the in vitro findings by confirming interactions with key protein targets associated with oxidative stress and metabolic pathways. **Conclusions:** Structural variation between species-derived alginates directly impacts their biological activities. TA represents a promising candidate for metabolic and anti-inflammatory therapies, while HA may be more suitable for neuroprotective interventions. These results emphasize the importance of source-specific alginate selection for developing targeted pharmaceutical applications.

## 1. Introduction

Marine macroalgae, especially brown seaweeds, have received great attention as a significant source of renewable bioactive compounds, which have different applications in therapies and biotechnology. *Turbinaria ornata* and *Hormophysa cuneiformis* are brown algae that are common in tropical and subtropical marine environments and characterized by their rich composition of polysaccharides, especially alginate [1,2]. Alginate is a predominant anionic polysaccharide that is found in the cell walls of several brown seaweeds, including *Turbinaria ornata*, *Hormophysa cuneiformis*, *Laminaria digitata*, and *Ascophyllum nodosum* [3]. Alginate’s structure is composed of linear copolymers of β-D-mannuronic acid (M) and α-L-guluronic acid (G), arranged in both homopolymeric (MM or GG) and heteropolymeric (MG) blocks [4].

There are several factors affecting alginate’s biochemical profile, including extraction techniques, algal species, environmental conditions, and geographic location. Although alginate has been extensively characterized from many temperate species, there is limited understanding of how Red Sea environmental conditions, characterized by high salinity, temperature, and light intensity, affect alginate composition and functionality. Thus, systematic comparative studies on Red Sea-derived alginates remain scarce, despite the unique ecological and biochemical properties of their algal sources.

Moreover, most previous studies have focused primarily on physicochemical characterization or single biological properties, such as antioxidant or antidiabetic effects. However, the integrated evaluation of multiple therapeutic potentials (antioxidant, anti-inflammatory, antidiabetic, neuroprotective, and hepatoprotective) in relation to structural variations has not been comprehensively investigated. This knowledge gap limits the ability to rationally select alginate sources for specific biomedical applications.

Structural variability of alginate determines its functional characteristics, thus making the species-specific characterization a vital factor. Similarly, both *T. ornata* and *H. cuneiformis* are endemic to the Red Sea and are observed to have significant promise as alginate sources. These potential characteristics of alginate are due to their substantial yield and diverse biological activity [5,6]. Previous investigations have declared the potential effect of alginate in controlling several signalling pathways, including inflammation, glucose metabolism, oxidative stress, and neurodegenerative pathways [7,8]. Alginate has a significant antioxidant activity by scavenging reactive oxygen species (ROS), such as oxygen radicals and hydroxyl radicals, thereby decreasing oxidative damage at the cellular level [8]. Also, alginate induces the expression level of the transcription factor Nrf-2 (Nuclear factor erythroid 2-related factor 2) and the antioxidant response element (ARE) Nrf-2/ARE signaling pathway, resulting in enhancement of the expression levels of Heme oxygenase-1 (HO-1) and superoxide dismutase 1 (SOD1) [9]. These characteristics reinforce the potential role of alginate in overcoming conditions related to oxidative stress, including neurodegeneration, diabetes, and liver damage.

Furthermore, alginate exerts a potential anti-inflammatory effect by targeting mediators of prostaglandin synthesis, including cyclooxygenase enzymes (COX-1 & COX-2) [10]. Alginate also has antidiabetic activity, which can modulate the glycemic rate by inhibiting α-amylase and α-glucosidase enzymes involved in carbohydrate digestion. The reduction in the postprandial glucose spike is a result of the delay in starch breakdown [11]. Alginate has a neuroprotective role and has an inhibition capacity for high expression levels of butyrylcholinesterase (BChE) in advanced Alzheimer’s disease [12]. In addition, alginate has a hepatoprotective role by reducing oxidative stress and stabilizing mitochondrial membranes [13].

In this context, the present study aims to fill this gap by isolating and comparing alginates extracted from two Red Sea brown algae, *H. cuneiformis* and *T. ornata*, to determine how their structural differences influence multifunctional biological properties. Unlike previously reported alginates from temperate species, these alginates exhibit distinct physicochemical signatures arising from their environmental adaptation, potentially leading to differential therapeutic performance.

This work contributes to the discovery of natural, safe, and sustainable biomaterials with pharmaceutical and nutraceutical relevance and supports the valorization of underutilized marine resources for biomedical applications.

## 2. Results

### 2.1. FTIR Characterization of Alginate Extracts

**Figure 1:**
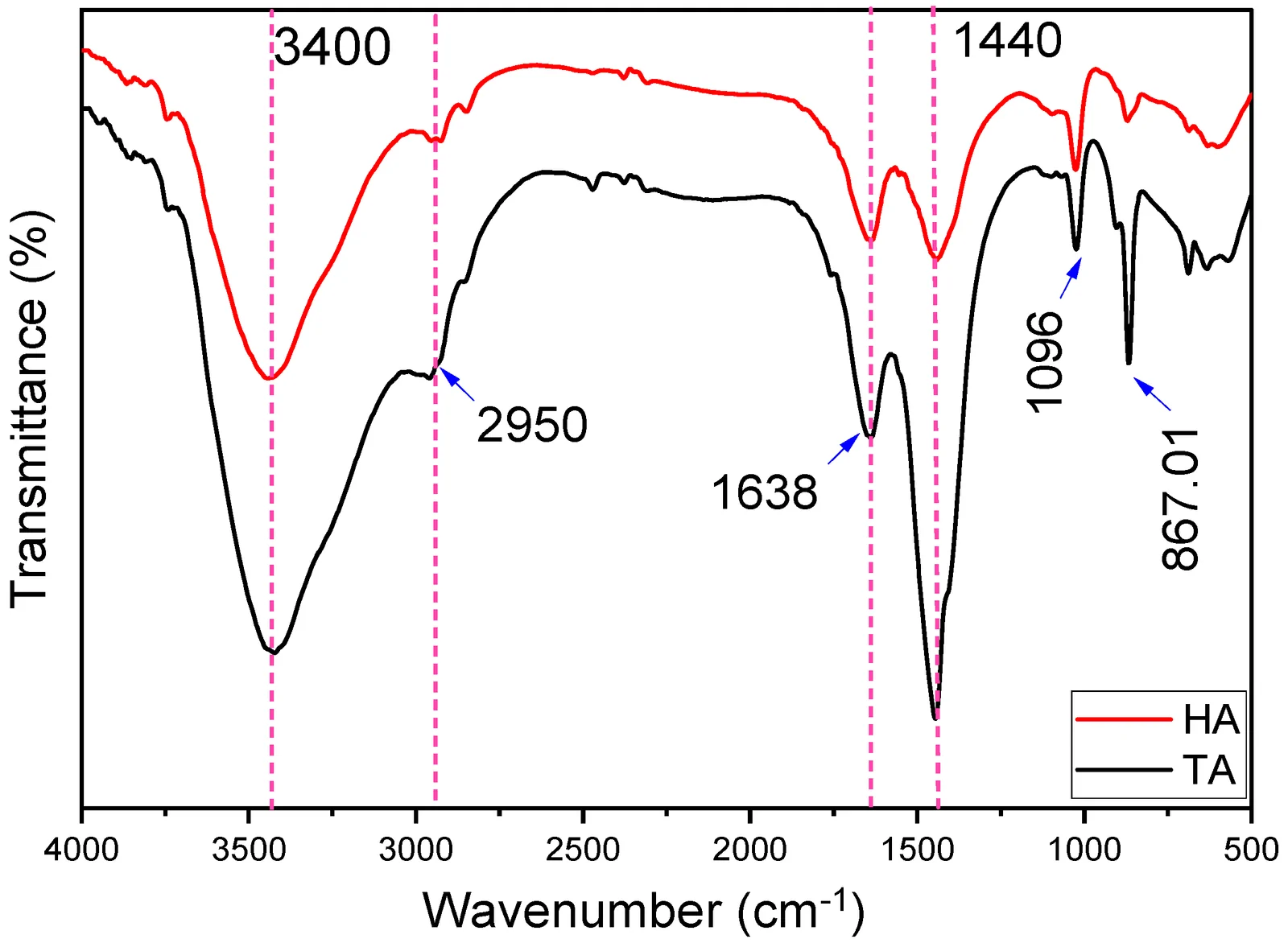
FTIR spectra of alginate extracted from *Hormophysa cuneiformis* (HA) and *Turbinaria ornata* (TA). Dashed lines indicate identical peak positions observed in both samples.

The FTIR spectra of alginate samples extracted from *H. cuneiformis* and *T. ornata* are presented in Figure 1. The spectra exhibit characteristic absorption bands confirming the presence of functional groups typically associated with alginate polysaccharides. A broad band observed around 3200–3400 cm^−1^ corresponds to the stretching vibrations of hydroxyl (-OH) groups, indicative of polysaccharide structures. This band reflects the hydrophilic nature of alginate and the extensive hydrogen bonding within its matrix.

A strong absorption peak in the range of 1600–1650 cm^−1^ represents the asymmetric stretching vibration of the carboxylate (-COO^−^) group, while the band around 1400–1450 cm^−1^ corresponds to the symmetric stretching of the same group. These signals confirm the presence of uronic acid residues, specifically guluronic and mannuronic acid units, which are the fundamental building blocks of alginate.

In the region between 1000–1100 cm^−1^, prominent peaks were detected, which can be attributed to C–O–C stretching vibrations of glycosidic linkages within the alginate polymer backbone.

Slight shifts in peak positions and variations in intensities between *H. cuneiformis* and *T. ornata* alginate spectra suggest differences in the mannuronic-to-guluronic acid (M/G) ratio, which may influence the physicochemical properties of the alginate. Additionally, subtle differences in the region below 900 cm^−1^ may reflect structural variations arising from the algal sources. The bands around 1625 cm^−1^ and 1030 cm^−1^ were more pronounced in HA, confirming the presence of characteristic alginate functionalities.

### 2.2. X-Ray Diffraction (XRD) Analysis of Alginate Extracted from Brown Algae

**Figure 2:**
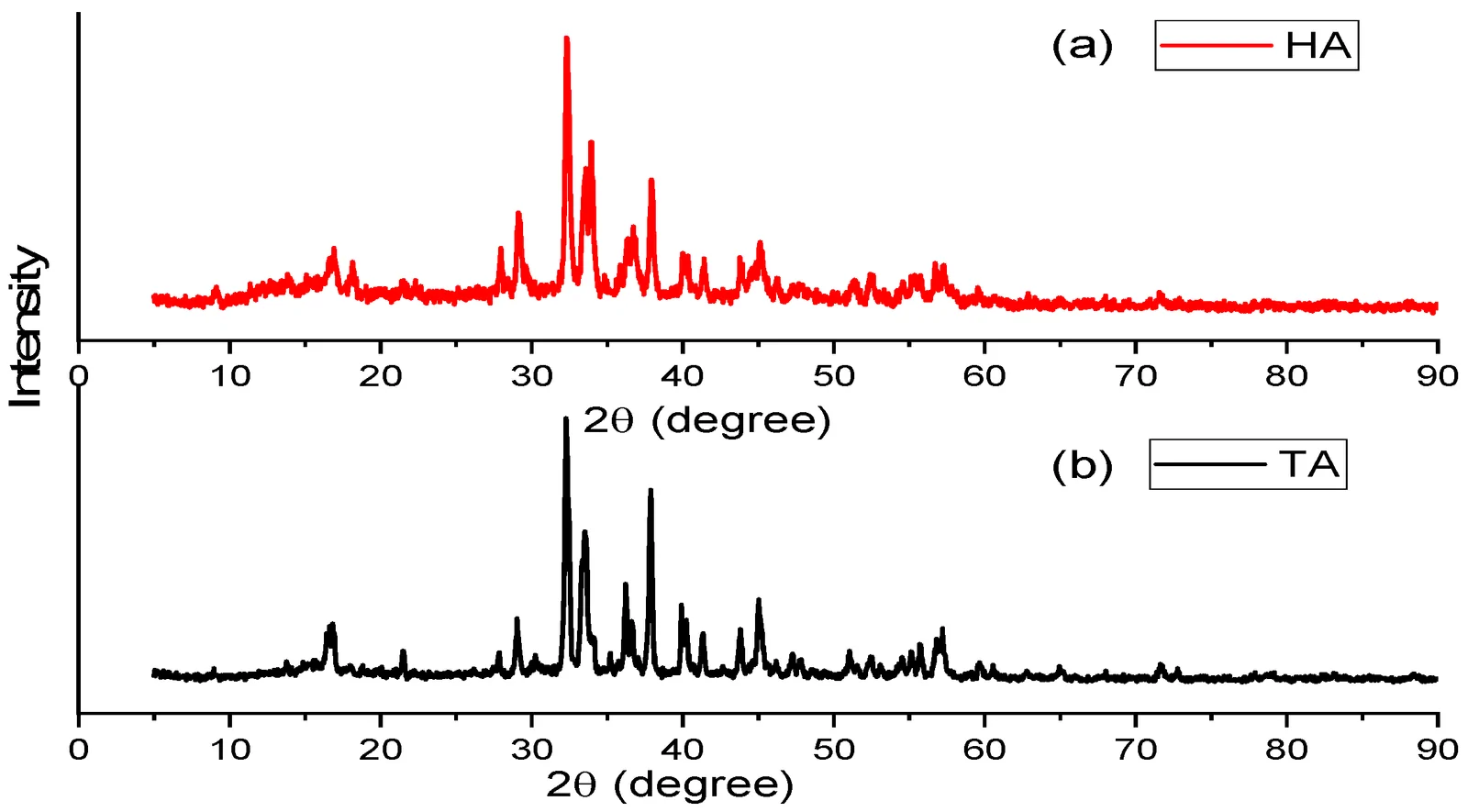
X-ray diffraction (XRD) patterns of alginate extracted from (**a**) *Hormophysa cuneiformis* and (**b**) *Turbinaria ornata*.

**Figure 3:**
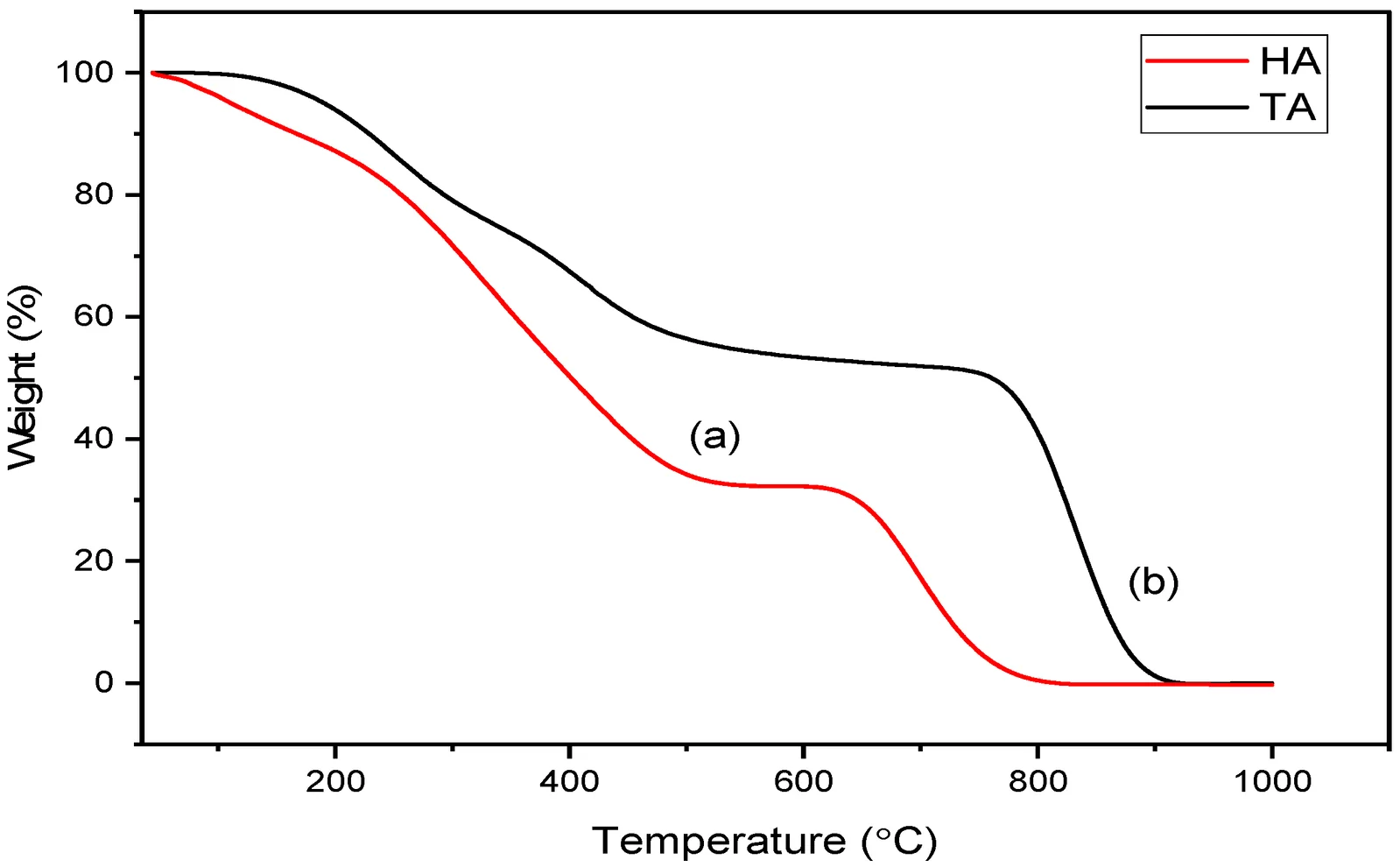
Thermogravimetric analysis (TGA) curves of alginate extracted from (**a**) *Hormophysa cuneiformis* (**b**) *Turbinaria ornata*, showing thermal decomposition behavior as a function of temperature.

The patterns of XRD of alginate samples, which are extracted from *Hormophysa cuneiformis* and *Turbinaria ornata*, are shown in Figure 2. Both alginate samples exhibited several diffraction peaks within the 2θ range of 10° to 60°, indicating a semi-crystalline nature typical of polysaccharides (alginate). It was observed that the appearance of distinct diffraction peaks within the 28–38° range shows the presence of partial crystalline domains, indicating a moderate level of molecular ordering in the alginate structure. Alginate samples, which were extracted from *Hormophysa cuneiformis* (HA), were observed to have slightly sharper and more intense peaks compared to alginate samples that were extracted from *Turbinaria ornata* (TA). These results show that HA has a relatively higher degree of crystallinity than TA, as shown in Figure 3a. Furthermore, these results could affect variations in molecular weight, the proportion of mannuronic (M) and guluronic (G) acid residues, or the extraction efficiency from the brown algal sources. However, the existence of crystalline domains, along with both patterns, exhibits large humps, especially obvious beyond 40°, indicating the amorphous nature of alginate. These broad characteristics propose the existence of disordered regions within the polymer matrix, which is popular in naturally derived polysaccharides. Moreover, the alginate from *Turbinaria ornata* seems to display a more amorphous profile than that from *Hormophysa cuneiformis*, which may affect its gelling behavior, mechanical strength, and other application-relevant characteristics. Overall, the XRD analysis reinforces that both alginate extracts exhibit a semi-crystalline structure. The diffractogram revealed partially ordered peaks indicating a semi-crystalline structure of the extracted alginate.

### 2.3. Thermal Stability and Decomposition Behavior of Alginates

The thermogravimetric analysis (TGA) of alginate extracted from *Hormophysa cuneiformis* and *Turbinaria ornata* showed obvious thermal degradation patterns that showed variations in their structural composition and thermal stability, as shown in Figure 3. Both alginate samples were exposed to several stages of the degradation process. Three major weight loss stages were detected in the HA sample. The first stage was observed to be between 45 °C and 167 °C, which corresponded to the evaporation of bound moisture and low molecular weight volatile compounds, with a modest weight loss of 8.95%. The 2nd stage was observed to be between 168 and 581 °C, which is reported for the most important degradation, involving the breakdown of alginate’s polysaccharide backbone and side chains, resulting in a 52.41% weight reduction. And the final decomposition stage was observed to be crossing from 582 °C to 996 °C, indicating carbonaceous residue degradation, with an additional 29.57% loss, resulting in a total weight loss of approximately 90.93% (Figure 3).

On the other hand, the initial stage was observed from 46 °C to 338 °C, with a considerably higher weight loss of 22.92%, indicating a higher content of volatile or thermally unstable constituents in the TA alginate sample. The 2nd stage was observed to be from 338 °C to 654 °C and exhibited a 20.42% weight loss, which can be attributed to the depolymerization of the alginate backbone and breakdown of uronic acid residues. And the final stage was observed from 655 to 989 °C, which indicated a substantial 47.95% mass loss (Figure 3). These thermal parameters are summarized in Table 1. It was observed that the total weight loss reached 90.79%, comparable to that of the HA sample. It was observed that HA had a relatively narrow first stage and more intense second-stage decomposition compared to TA Alginate. This indicates that the HA sample is a more thermally stable and homogeneously structured alginate than the TA sample. At the same time, the broader and earlier degradation onset in the TA sample may indicate more heterogeneous or loosely bound molecular domains. These thermal behaviors highlight potential variations in the physicochemical construction of the alginate samples, likely influenced by their algal source.

### 2.4. Elemental Composition Analysis of the Extracted Alginate

The basic chemical composition of alginate extracted from *H. cuneiformis* and *T. ornata* was evaluated using elemental analysis. The data results are presented in Table 2 and offer insight into the organic structure and possible functional constituents of each alginate sample. It was observed that the carbon content was relatively similar between the two Alginate samples, with HA containing 11.98% and TA slightly lower at 11.19%. This consistency indicates that both alginate samples have a comparable carbohydrate backbone, as carbon is the primary component of polysaccharide structures. It was observed that hydrogen levels were also close, with TA showing a slightly higher value (2.29%) compared to HA (2.16%). This small difference could be close to differences in hydrogen bonding or hydration levels in the polysaccharide chains. It was also observed that TA (5.19%) exhibited a significantly higher nitrogen content compared to HA (2.69%).

Furthermore, TA (1.63%) had a higher sulfur content than HA (0.97%). In general, nitrogen and sulfur content variations between HA and TA indicate that there are functional variation behaviors between the two alginate samples, depending on their elemental profiles. Overall, these results emphasize the effect of algal species on the biochemical composition of extracted alginates and their biochemical functions.

### 2.5. Monosaccharide and Uronic Acid Composition of Alginate by HPLC

Alginate samples were exposed to high-performance liquid chromatography (HPLC) to determine the monosaccharide and uronic acid composition. Data showed that differences in sugar composition indicate that there are structural and biological variations between the two alginate sources, as shown in Table 3. The HA sample was observed to have a significantly higher concentration of neutral sugars (glucose) compared to the TA sample, which was 11.32 µg/g and 2.84 µg/g, respectively. It was observed that galactose and rhamnose were exhibited in both samples, but were more abundant in HA (7.74 and 4.78 µg/g, respectively) than in TA (6.37 and 3.55 µg/g). These sugars are typically accompanied by structural side chains or branching in polysaccharides, exhibiting a slightly more complex sugar profile in the HA sample. It was also observed that there was a higher concentration of fucose in the TA sample compared to the HA sample, at 4.71 µg/g and 3.23 µg/g, respectively. Fructose was only detected in the HA extract (4.02 µg/g), revealing a unique sugar profile in this species that the biochemical composition of Hormophysa may influence. Besides basic sugar analysis, uronic acid content was also observed, which is an important element in the composition of alginate. Data revealed that HA exhibited 10.69 µg/g of uronic acids, whereas TA displayed a slightly elevated level of 11.85 µg/g. Overall results indicate that differences in both monosaccharide composition and uronic acid levels between HA and TA suggest species-specific differences in alginate structure. These quantitative sugar variations highlight structural differences between the two alginate sources.

### 2.6. Computational Analysis

**Figure 4:**
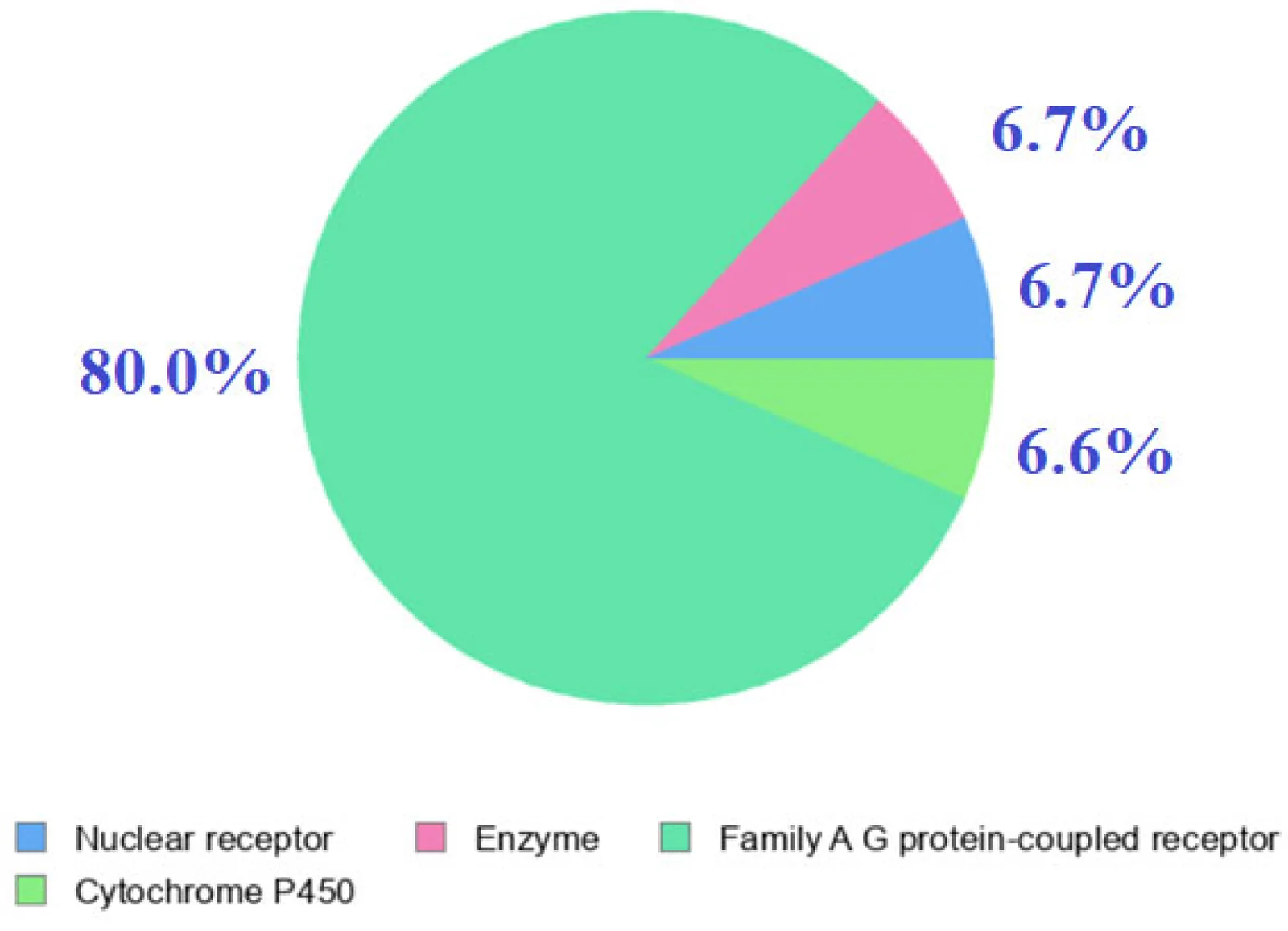
Regulatory cellular classes targeted by alginate.

**Figure 5:**
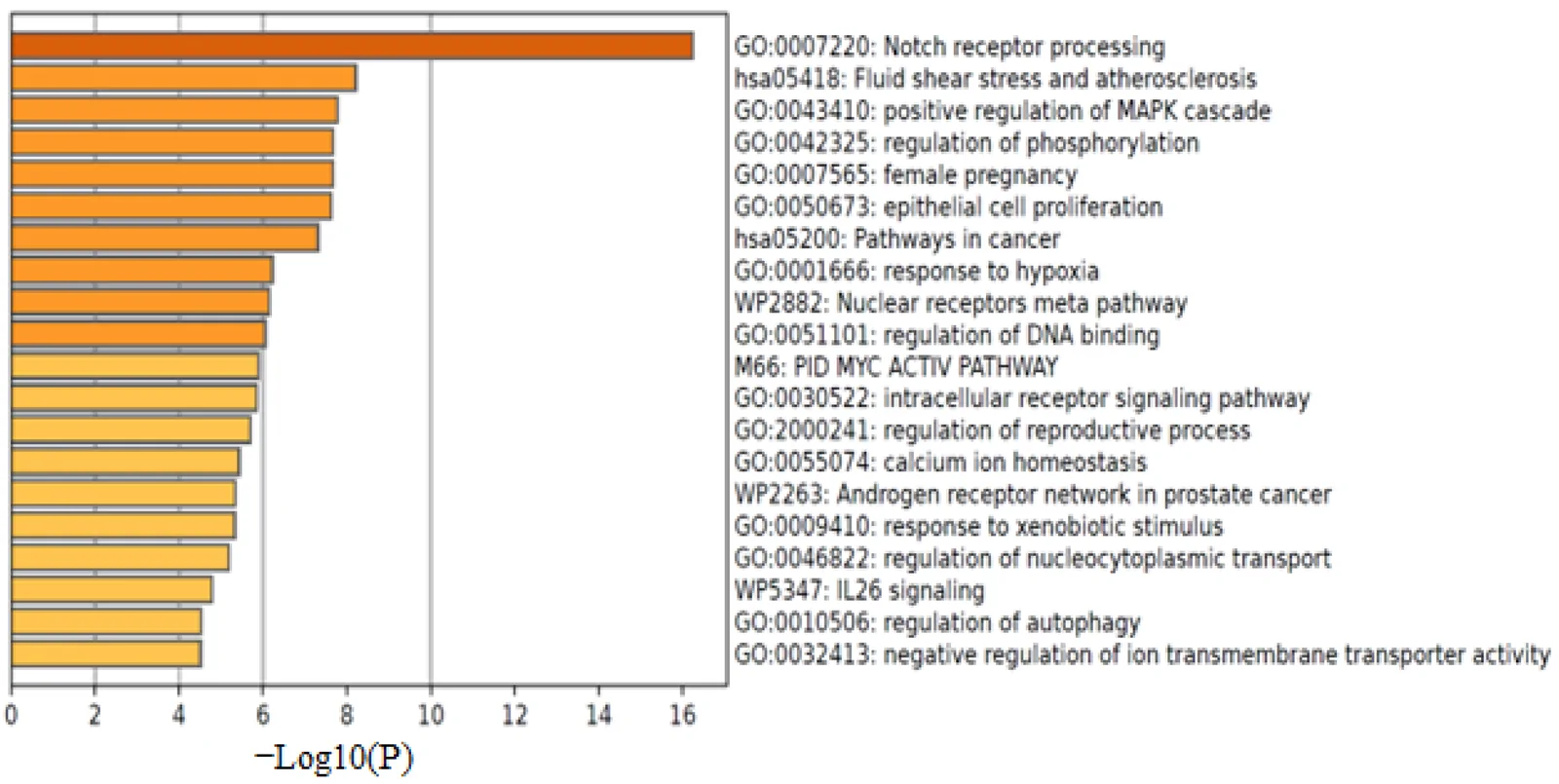
Key cellular events and Gene Ontology (GO) biological processes associated with alginate treatment were analyzed using Metascape.

Computational results demonstrated valuable insights into the multi-target potential of alginate extracts. Swiss Target Prediction has been employed to detect potential human protein targets of alginate by using a SMILES-based query. The output list included several protein target classes. Particularly, G-protein-coupled receptors (GPCRs) dominated the majority of predictions (80% of hits), with the remainder distributed amongst ion channels, enzymes, and signalling transporters. Results of Target Prediction showed several alginate-gated ion channels interaction (glycine receptor α1 GLRA1, UniProt P23415; GLRA2 P23416) and a voltage-gated potassium channel (HERG/KCNH2, Q12809) were predicted targets as shown in Table 4 & Figure 4. Similarly, ATP-dependent transporters such as the bile salt export pump (ABCB11) (O95342) are included in the list. The highest scoring hits were observed in the predicted butyrylcholinesterase (BChE; UniProt P06276) and pancreatic α-amylase (UniProt P04746) targets. Among the enzyme-related targets predicted by Swiss Target Prediction, additional manually curated hits corresponding to hydrolase and oxidoreductase families (e.g., butyrylcholinesterase [P06276], pancreatic α-amylase [P04746]) were incorporated into the docking study to validate potential mechanisms of antioxidant, antidiabetic, and neuroprotective action. Overall, in conclusion, the results showed that alginate can target all major drug target families, including oxidoreductases and hydrolases (enzymes), nuclear receptors, ion channels, and ABC-type transporters, which include significant mammalian drug targets. The set of predicted targets for functional enrichment was investigated using Metascape, as shown in Figure 5. Gene Ontology (GO) is used to predict the target biological process through alginate, including oxidation–reduction process, metabolic process, cellular response to oxidative stress, and inflammatory response. It was observed that the GO:0055114 (oxidation–reduction process) and GO:0006954 (inflammatory response) were highly ranked, indicating that alginate’s targets are involved in immune signaling pathways and redox regulation. Based on Gene Ontology (GO) and pathway enrichment analyses (Metascape and GeneMANIA), alginate-associated targets were mainly linked to oxidation–reduction, inflammatory, and metabolic regulatory processes. The most significantly enriched pathways included Nrf2/HO-1 antioxidant signaling, NF-κB-mediated inflammatory regulation, and PI3K/Akt signaling, which are closely associated with oxidative stress response, cellular protection, and metabolic homeostasis. Additional enriched pathways, such as MAPK cascade, IL-26 signaling, and Notch receptor processing, further support alginate’s multi-target therapeutic potential.

**Figure 6:**
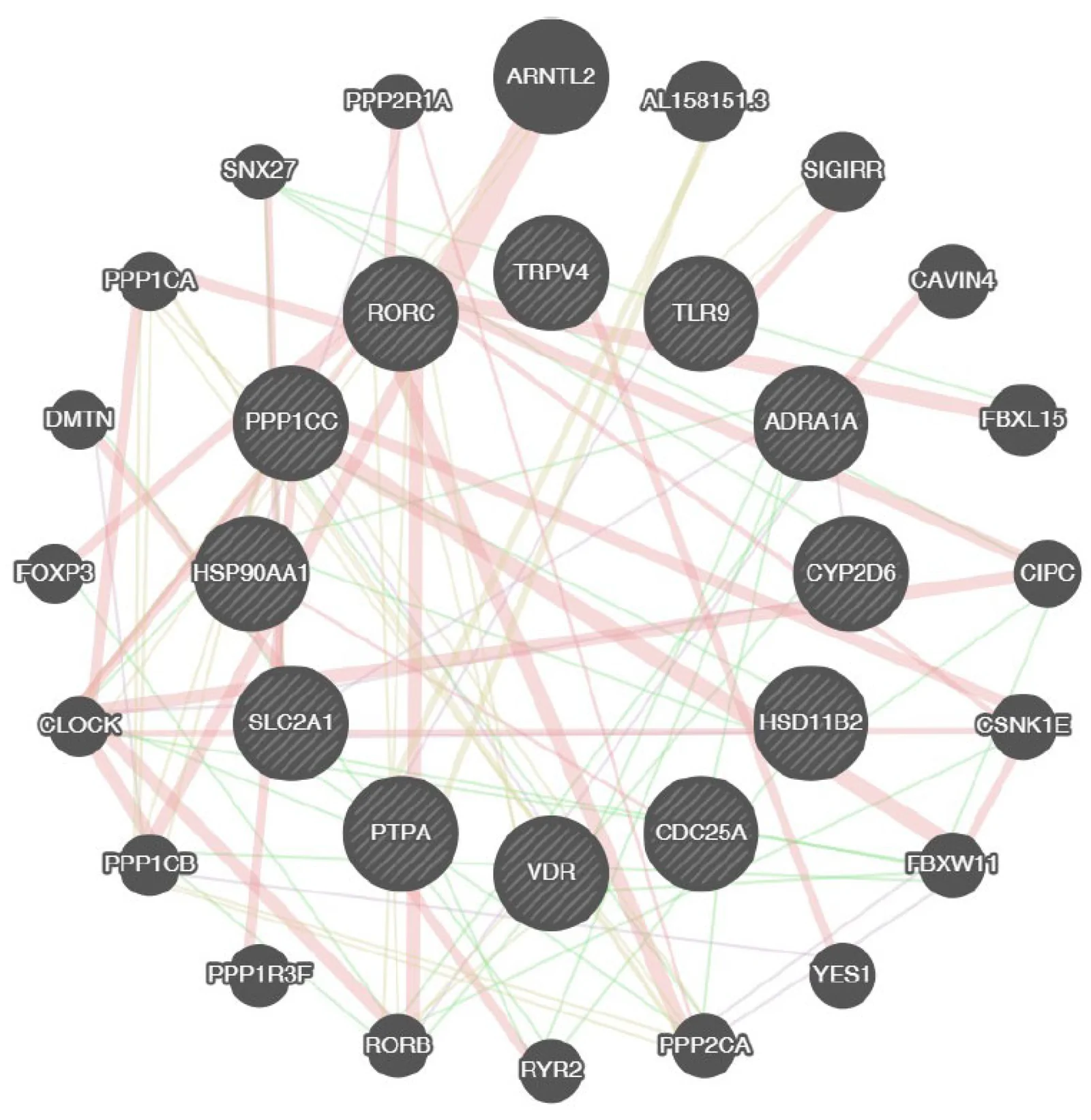
Gene-gene interactions and colocalization of genes affected by Alginate predicted by GeneMANIA.

GeneMANIA is used to explore the genes associated with their interaction network with alginate, as shown in Figure 6. The GeneMANIA network investigates the major interconnections, particularly antioxidant enzymes such as superoxide dismutase 1 (SOD1) and Glutathione peroxidase 4 (GPX4) clustered together, indicating their shared role in reactive oxygen species detoxification, and butyrylcholinesterase (BChE) and cyclooxygenase-1 (COX-1) in liver tissues. In conclusion, the network indicates that alginate’s target pathways are oxidative stress defense and inflammatory signaling pathways.

Molecular docking was accomplished for alginate (G-M dimer) against several proteins, including BChE (PDB: 1P0M), pancreatic α-amylase (PDB: 1b2y), COX-1 (PDB: 6Y3C), Cu/Zn-SOD1 (PDB: 5YTO), and GPX4 (PDB: 5H5Q), as shown in Table 5. Also, there was a comparative analysis between alginate and docked standard inhibitors, including rivastigmine or donepezil for BChE, acarbose for α-amylase, aspirin for COX-1, and Vitamin C for GPX4, to investigate the inhibitory effect of alginate. It was observed that alginate interacts with specific amino acids in each enzyme’s active site, but with the same or moderate binding affinity.

The results of docking analysis displayed that alginate could interact with key amino acids (HIS 438 ionic) in the binding site of butyryl cholinesterase (PDB: 1P0M) with a docking score of −7.5459 kcal/mol, which is substantially higher than −6.69 kcal/mol for the control drug rivastigmine. It was observed that alginate occupied the catalytic cleft of the human pancreatic alpha-amylase (PDB: 1B2Y) with key amino acids including Asp197, HIS 305, and Asp300. In the α-amylase pocket, alginate made fewer contacts and scored only −6.5440 kcal/mol compared with control or acarbose’s −8.8135 kcal/mol (acarbose’s multi-glycosidic scaffold forms additional H-bonds with Arg195, Asn298).

Alginate is observed to form two hydrogen bonds with key catalytic amino acids, including GLU 524, PRO 84, ARG 120, and ARG 83, with docking energy −6.1609 kcal/mol in the COX-1. It was observed by comparison that alginates had higher docking energy than control or aspirin (−4.8185 kcal/mol), which acetylates Ser530 in the active site of COX-1.

Furthermore, alginate interacts with key amino acids, including GLY 10, ASN 53 and LYS 9 in the metal-binding loop of SOD1, with a binding score of −5.0492 kcal/mol, which was higher than the docking energy of control Vitamin C (−3.91 kcal/mol).

Alginate was observed to bind to HIS 42, ARG 60 in the active site of GPX4 with a docking score of −4.7917 kcal/mol, while Vitamin C had a docking score of −3.7975 kcal/mol. Overall, in conclusion, the docking score of alginates was similar to that of standard reference ligands across all target proteins. Also, alginate showed a moderate binding affinity with COX-1, SOD1, and GPX4, revealing its inhibition potential to regulate inflammatory and oxidative stress pathways.

### 2.7. Butyl Choline Esterase Inhibitory Activity Assay

**Figure 7:**
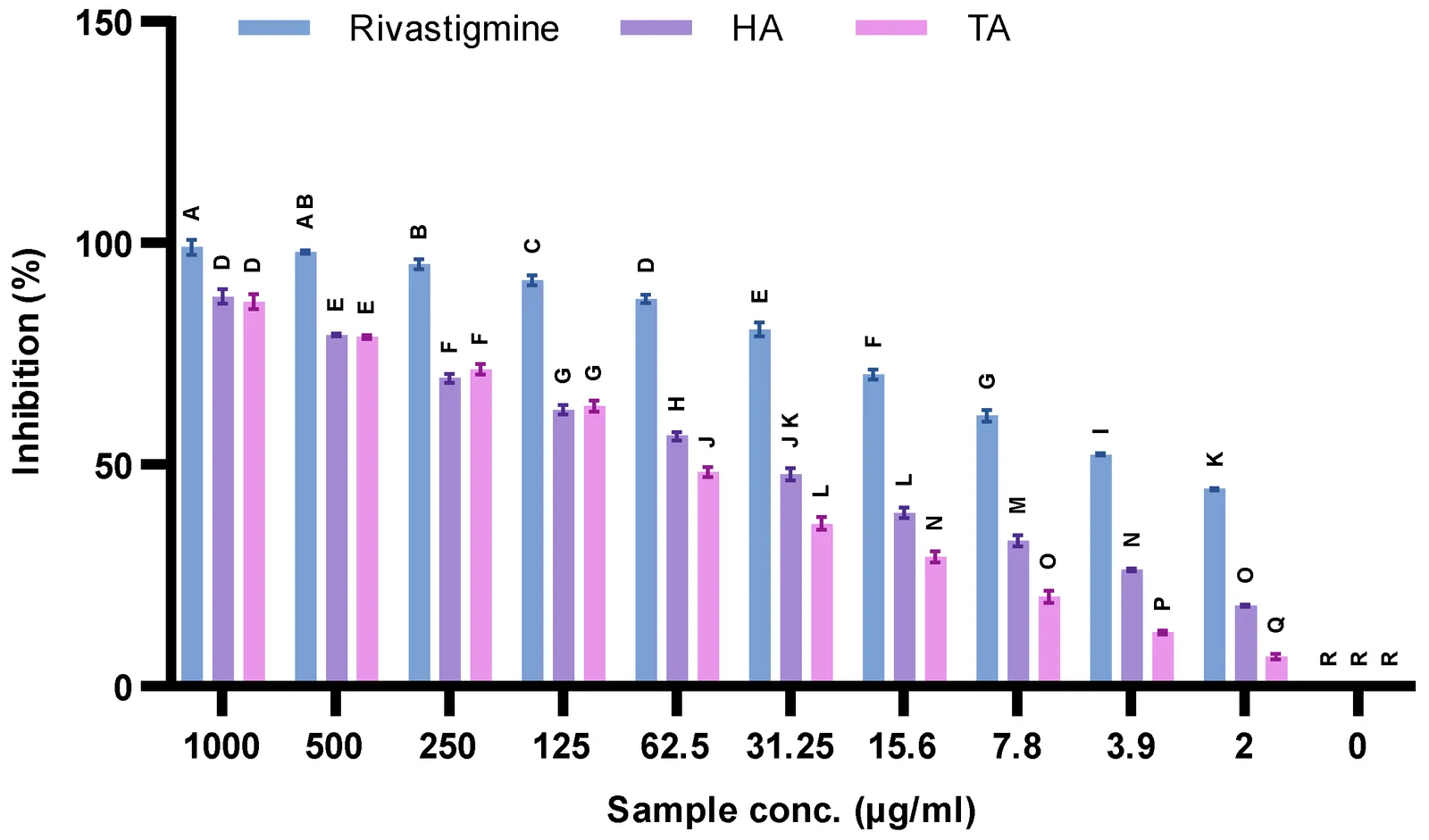
Inhibition of butyryl choline esterase (BChE) by alginate extracted from *Hormophysa cuneiformis* and *Turbinaria ornata* compared with rivastigmine. Data are presented as mean ± SD (n = 3). Different letters above the bars indicate statistically significant differences among groups according to one-way ANOVA followed by Tukey’s post hoc test (*p* < 0.05).

Figure 7 revealed the results of assessing the inhibitory effects of alginate extracted from *Hormophysa cuneiformis* and *Turbinaria ornata* against butyrylcholinesterase enzyme compared to the reference control, rivastigmine. The results of enzyme inhibition (%) were expressed as a percentage and IC_50_. IC_50_ is defined as the concentration of alginate required to inhibit 50% of the Butyl Choline Esterase Enzyme. The compound, which has the strongest level of inhibitory activity, has the lowest IC_50_. Butyl choline esterase inhibition activity has improved gradually with increasing concentration of the extract. The results in Figure 7 revealed that alginate from *Hormophysa cuneiformis* had stronger inhibitory activity (IC_50_ = 39.01 µg/mL) than that from *Turbinaria ornata* (IC_50_ = 107.38 µg/mL) and was observed to be less effective than rivastigmine (IC_50_ = 3.6 µg/mL). These results revealed that HA may be more significant in the management of neurodegenerative conditions such as Alzheimer’s disease.

### 2.8. Anti-Inflammatory Activity

**Figure 8:**
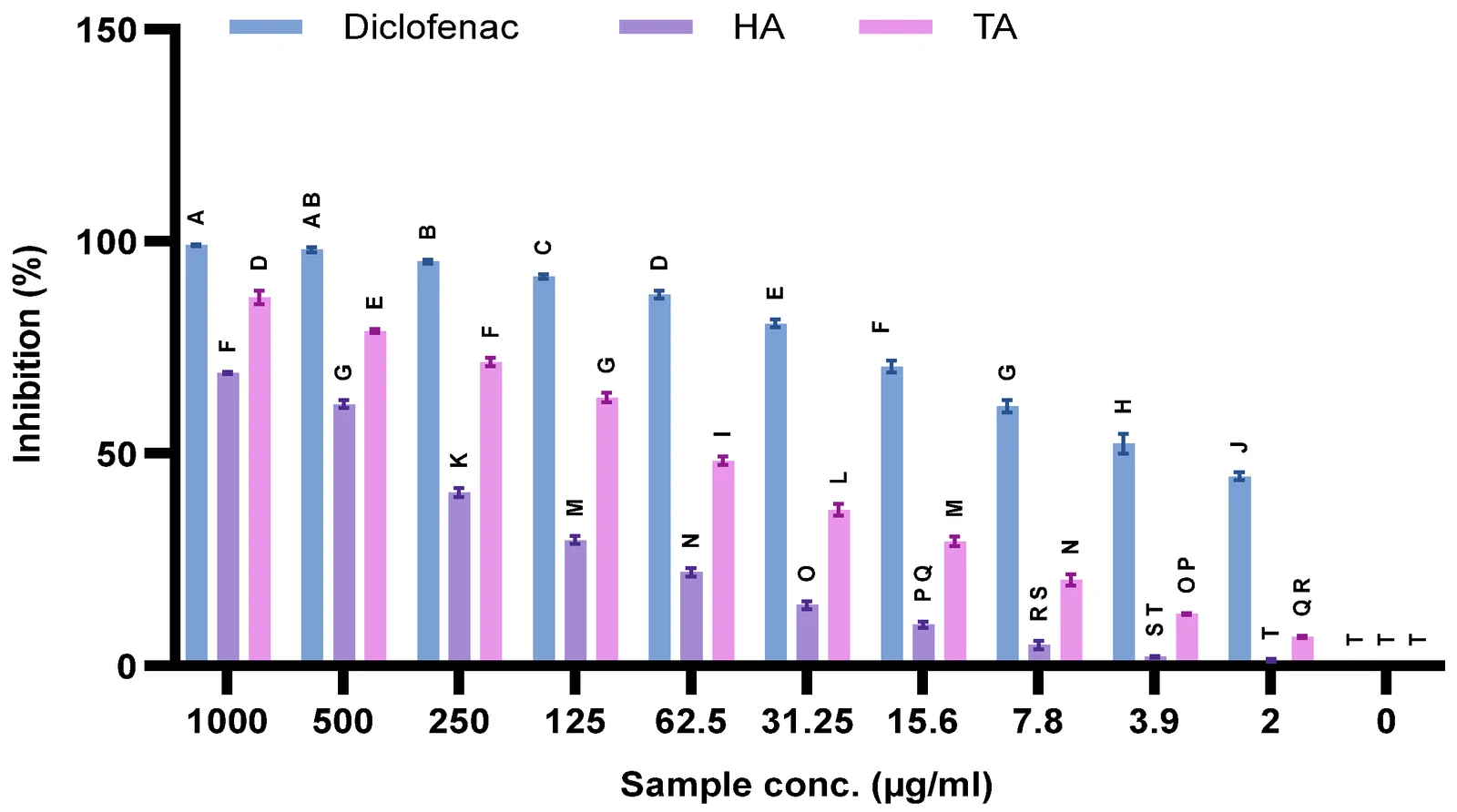
Inhibition of COX-1 activity by alginate extracted from *H. cuneiformis* (HA) and *Turbinaria ornata* (TA) compared with Diclofenac. Data are presented as mean ± SD (n = 3). Different letters above the bars indicate statistically significant differences among groups according to one-way ANOVA followed by Tukey’s post hoc test (*p* < 0.05).

Figure 8 shows the results of assessing the inhibitory effects of alginate extracted from *H. cuneiformis* and *T. ornata* against the COX-1 enzyme compared to the reference control diclofenac. COX-1 inhibition (%) is expressed as IC_50_, which is defined as the concentration of alginate that is required to inhibit 50% of the COX-1. The data exhibited that TA displayed significantly high inhibition activity with IC_50_ = 69.61 µg/mL compared to HA (IC_50_ = 360.22 µg/mL). However, both alginate extract samples have notably less inhibition activity than diclofenac (IC_50_ = 3.63 µg/mL). TA was observed to have a significant anti-inflammatory characteristic. The COX-1 inhibition activity of alginate was observed to increase in a dose-dependent manner.

### 2.9. Antidiabetic Activity

**Figure 9:**
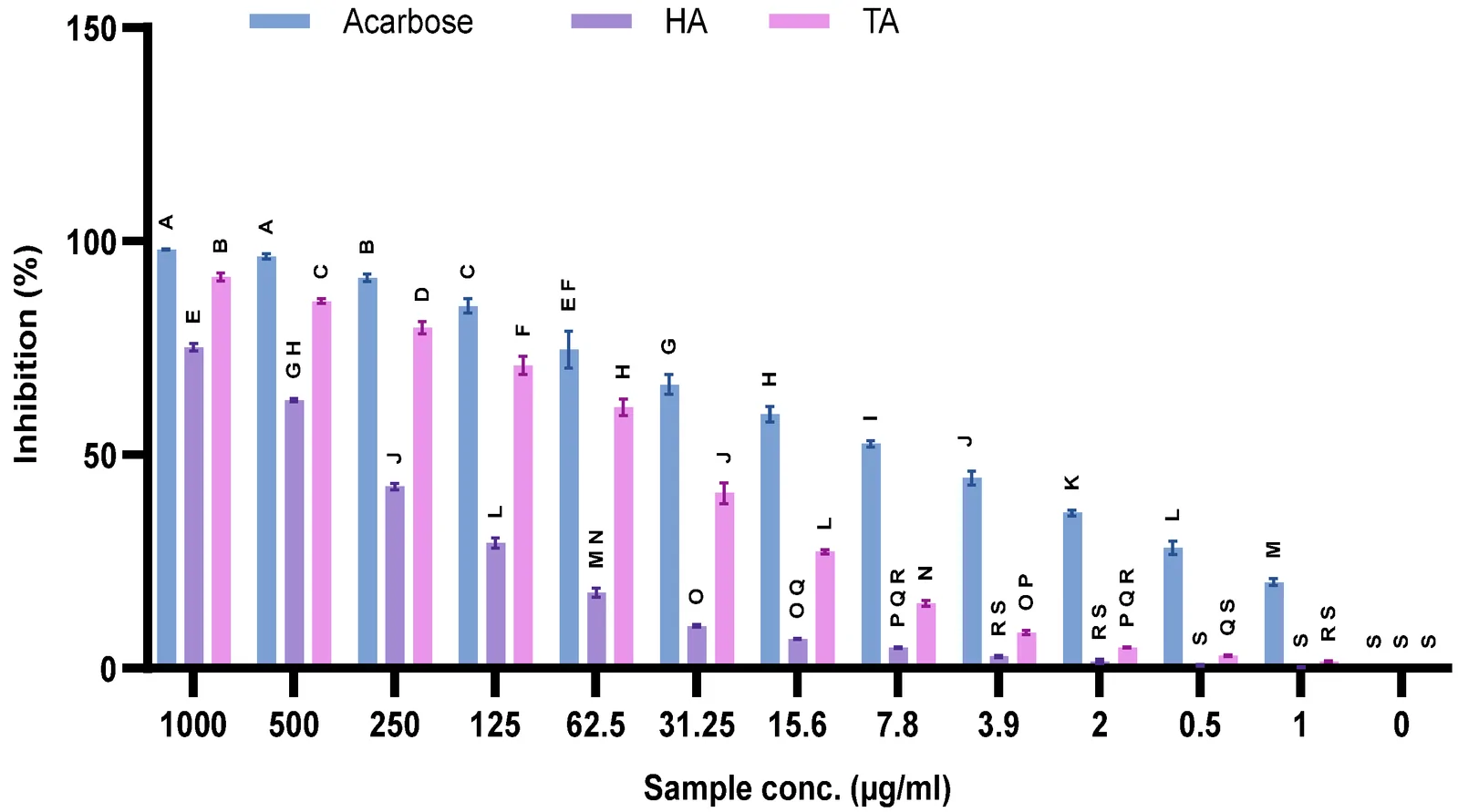
Inhibition of α-amylase activity by alginate extracted from *H. cuneiformis* (HA) and *T. onata* (TA) compared with Acarbose. Data are presented as mean ± SD (n = 3). Different letters above the bars indicate statistically significant differences among groups according to one-way ANOVA followed by Tukey’s post hoc test (*p* < 0.05).

Figure 9 exhibited the results of evaluating the inhibitory effects of alginate extracted from *H. cuneiformis* and *T. ornata* against α-amylase enzyme by comparing with acarbose as a standard drug. The results exhibited that alginate had an inhibitory effect against α-amylase enzyme activity in all concentrations. α-amylase enzyme activity inhibition (%) is expressed in IC_50_, which is defined as the concentration of alginate required to inhibit 50% of the α-amylase enzyme. The compound, which has the strongest level of inhibition activity, has the lowest IC_50_. Results showed that TA had a significant antidiabetic potential more than HA, with IC_50_ 45.14 µg/mL and 341.48 µg/mL, respectively. However, both TA and HA had an inhibitory activity that remained less than that of acarbose (IC_50_ = 6.52 µg/mL). It was also observed that α-amylase inhibition increased with rising extract concentrations. Overall, in conclusion, T. Alginate exerts an effective antidiabetic effect by reducing postprandial blood glucose levels.

### 2.10. DDPH Scavenging

**Figure 10:**
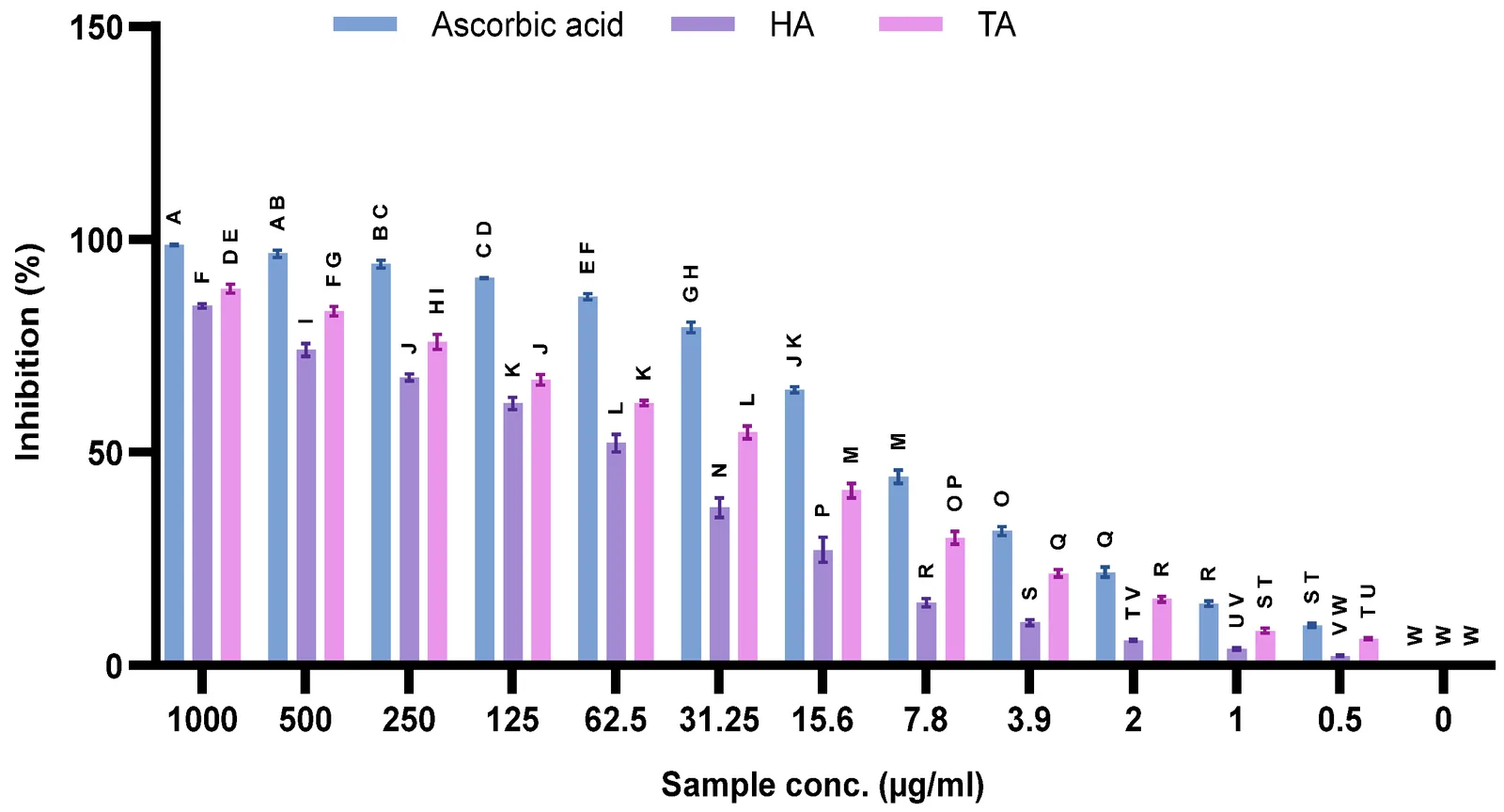
Inhibition of DPPH activity by alginate extracted from *H. cuneiformis* (HA) and *T. onata* (TA) compared with Ascorbic acid standard. Data are presented as mean ± SD (n = 3). Different letters above the bars indicate statistically significant differences among groups according to one-way ANOVA followed by Tukey’s post hoc test (*p* < 0.05).

The results in Figure 10 showed significant DPPH scavenging activity of alginate extracted from *H. cuneiformis* and *T. ornata*. From the results of the antioxidant activity assay, it was observed that TA had a significantly lower IC_50_ value (25.89 µg/mL) compared to HA (58.22 µg/mL), showing superior antioxidant capacity compared to ascorbic acid. This indicates that TA had a high efficacy in scavenging free radicals. It was observed that the antioxidant activity of alginate samples increased in a dose-dependent manner. Concerning the antioxidant activity assessed by the DPPH method and expressed as a percentage and IC_50_, which is defined as the concentration of extract required to scavenge 50% of the DPPH radical. The compound that had the strongest level of antioxidant activity had the lowest IC_50_.

### 2.11. Hepatoprotective Activity by Alginate Extracted from Hormophysa cuneiformis and Turbinaria ornata

**Figure 11:**
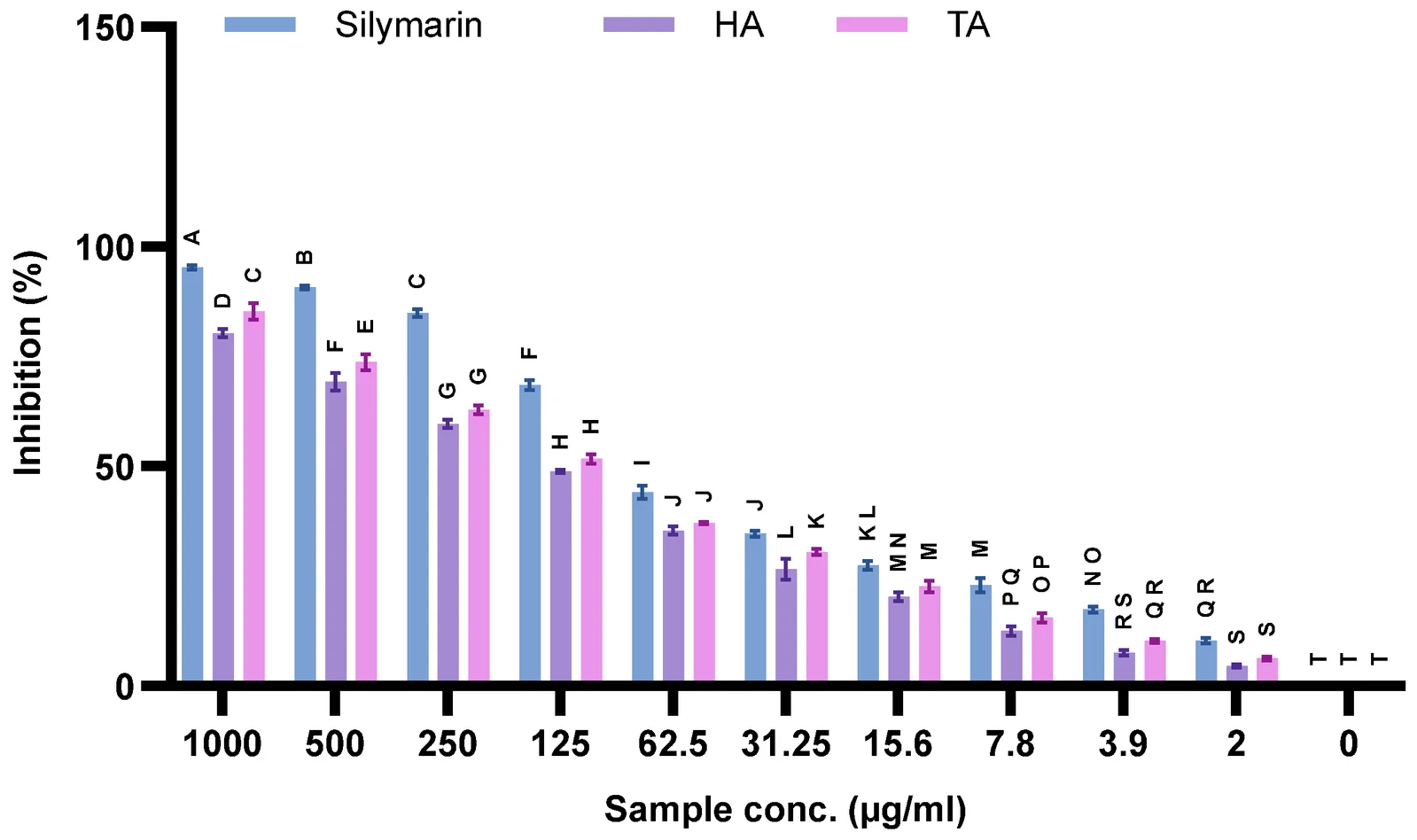
Inhibition of α-amylase activity by alginate extracted from *H. cuneiformis* (HA) and *T. onata* (TA) compared with Silymarin. Data are presented as mean ± SD (n = 3). Different letters above the bars indicate statistically significant differences among groups according to one-way ANOVA followed by Tukey’s post hoc test (*p* < 0.05).

The results in Figure 11 assess the hepatoprotective activity of alginate extracted from *Hormophysa cuneiformis* and *Turbinaria ornata*, compared with Silymarin, which was used as the standard drug. It was observed that HA cuneiform had the strongest protective effect at 1000 µg/mL, achieving 80.35% protection and an IC_50_ value of 138.36 µg/mL, indicating strong hepatoprotective potency. Also, TA exhibited a maximum protection of 85.35% at the same concentration, with a lower IC_50_ value of 118.21 µg/mL. Both TA and HA showed dose-dependent activity, with TA showing enhanced protection (IC_50_ = 118.21 µg/mL) over HA (IC_50_ = 138.36 µg/mL). After comparing with silymarin, it was observed that silymarin was more effective with an IC_50_ of 77.65 µg/mL. It could be observed that the hepatoprotective activity increases with increasing concentration of the extract.

In general, the correlation between docking affinities and in vitro IC_50_ values was observed across the tested enzymes. Specifically, alginate exhibited moderate docking scores (−6.1609, −7.5459, and −6.5440 kcal/mol) that aligned with its moderate inhibitory IC_50_ values for COX-1, BChE, and α-amylase (69.61 µg/mL, 39.01 µg/mL, and 45.14 µg/mL, respectively), confirming a consistent trend between computational and experimental findings. This relationship supports the predictive validity of the docking approach, where higher binding affinities corresponded to lower IC_50_ values and stronger biological inhibition.

### 2.12. Comparative Potency Between Alginate Extracted from Hormophysa cuneiformis and Turbinaria ornata

The results in Table 6 show that TA has superior bioactivity in anti-inflammatory, antidiabetic, antioxidant, and hepatoprotective assays. On the other hand, HA exhibited a distinct advantage in neuroprotective potential via stronger BChE inhibition. These variations suggest that while both alginate types have significant therapeutic characteristics, their applications may be targeted to HA in neurodegenerative conditions and TA in metabolic and inflammatory disorders.

## 3. Discussion

Alginate is a polysaccharide that occurs naturally in brown seaweeds, such as *Ascophyllum*, *Laminaria*, and *Macrocystis* species [4,14]. Alginate is found structurally in 2 forms, including homopolymeric blocks (M-blocks and G-blocks) or alternating sequences (MG-blocks), which comprise linear copolymers of β-D-mannuronic acid (M) and α-L-guluronic acid (G) residues. This unique composition reveals that alginate has distinctive physicochemical characteristics [4].

The present study shows investigations and bioassays of characterized alginate extracted from two brown algal sources, *H. cuneiformis* and *T. ornata*, and assesses their structural profile, biochemical, and pharmacological applications. Results revealed that the substantial source-dependent variability in elemental composition, physicochemical characteristics, and potential bioactivities suggests distinct therapeutic potential and applications for both alginates.

FTIR spectra analysis emphasized the presence of key functional groups, hydroxyl (-OH) and carboxylate (-COO−), that are typical of alginate polysaccharides. These functional groups, which are considered essential for water solubility and ionic cross-linking, define the backbone characteristics of alginate polymers. The intensity of the bands at 1600–1650 cm^−1^ and 1400–1450 cm^−1^ was slightly higher in HA, suggesting a greater content of guluronic residues. Similar quantitative peak variations in brown algal alginates [15].

The minor spectral differences observed between HA and TA, particularly in the COO^-^ stretching region, suggest a variation in the distribution of mannuronic (M) and guluronic (G) units. These structural differences influence polymer characteristics such as viscosity, gel-forming ability, and ion-binding properties, as reported previously for alginates with diverse M/G block arrangements [16,17]. The enhanced band intensities at 1600–1650 cm^−1^ and 1400–1450 cm^−1^ suggest that HA is relatively richer in G-blocks than TA. However, additional structural evaluation is still required for precise confirmation of the M/G distribution.

XRD patterns analysis confirmed these differences, with HA displaying more distinct and intense diffraction peaks, showing a comparatively higher degree of crystallinity than TA. These characteristics, including its molecular order and high proportion of G-blocks, improve its thermal and mechanical stability and facilitate stronger intermolecular associations. These results agreed with Zhang et al. [18,19]. This semi-crystalline pattern agrees with the results obtained for alginate extracted from other brown algae [20].

Furthermore, TGA profile analysis emphasizes these structural interpretations. HA exhibited a delayed onset of thermal degradation and greater thermal resistance, indicating a strong, compact, and stable polymeric network. Three phases of decomposition patterns monitored in both samples are consistent with prior reports on the thermolysis of natural polysaccharides in brown algae, with the first weight loss due to water evaporation, followed by backbone depolymerization and final degradation of residual carbonaceous material. TA has a larger and earlier thermal degradation, associated with its relatively lower crystallinity and potentially higher content of unstable, sulfated, or nitrogenous residues. These results are consistent with the observations described by Osman et al. [21] and Rashedy et al. [22]. The TGA profiles indicate different thermal behaviours for the two alginate samples: HA shows a pronounced major decomposition stage (168–581 °C) with a large mass loss, whereas TA exhibits a broader and earlier weight loss distribution (notably 22.9% below 338 °C) and a larger final decomposition stage. These patterns suggest that HA possesses a more homogeneous polymeric network while TA contains more heterogeneous or labile constituents, which is consistent with previous reports on natural algal polysaccharides [23].

Elemental analysis emphasized noticeable variations between the two alginate samples, particularly in nitrogen and sulfur content, both of which were mainly higher in TA. This shows the presence of proteinaceous residues or sulfated fucans, which were co-extracted with alginate. These results are consistent with the observations described by Jönsson et al. [24] and Ross et al. [25], who demonstrated that these residues can promote biological activities, mainly those related to redox homeostasis and inflammation modulation. Monosaccharide profiling analysis by HPLC showed that HA had higher levels of glucose, galactose, and rhamnose, neutral sugars often included in branching and side-chain functionalities. This outcome aligns well with the results reported by Sun [26]. At the same time, TA displayed high levels of fucose and uronic acids, components typically related to bioactive fucoidan-like compounds. These monosaccharide compositions are consistent with those reported for alginate polysaccharides characterized in previous studies. Similar observations have been described by Xiao et al. [15]. These results aligned with Donati and Christensen [27], who demonstrated that the slightly higher uronic acid content in TA also corroborates its enhanced gelation potential and ion-binding capacity. The structural differences highlighted by FTIR and HPLC analyses appear to influence the biological responses of the two alginate samples. The higher G-block content and sulfur enrichment in TA likely contribute to its pronounced antioxidant, anti-inflammatory, and antidiabetic activities, whereas the more homogeneous network in HA supports stronger cholinesterase inhibition. These structural–functional relationships provide a clear rationale for the observed differences in the biological activities of HA and TA.

Neurodegenerative disorders such as Alzheimer’s disease are associated with cholinergic deficits, w29 [28]. This assay is used to evaluate the ability of alginate to prevent the activity of the enzyme butyrylcholinesterase, which hydrolyzes choline-based esters such as butyrylcholine [29]. Alzheimer’s disease is characterized by high levels of BChE activity in the later stages as a compensatory mechanism for downregulating the activity and function of acetylcholinesterase (AChE) [30]. One of the aims of this study is to explore whether TA and HA may improve cholinergic neurotransmission by inhibiting the breakdown of acetylcholine and its analogs, thereby enhancing memory and cognitive function. It was observed that HA displayed a superior activity against butyrylcholinesterase (BChE), with an IC_50_ of 39.01 µg/mL compared to 107.38 µg/mL for TA. This indicates that HA may be effectively appropriate for applications targeting neurodegenerative diseases such as Alzheimer’s, where BChE inhibition plays a significant therapeutic role. These results align with Jia et al. [31], who highlighted BChE inhibition as a promising target for brain-memory-enhancing alginate. In this study, docking of alginate, a biologically active polysaccharide, was performed to gain a theoretical understanding of its possible interactions with key therapeutic enzymes (COX-1, α-amylase, BChE, SOD1, and GPX4) related to inflammation, diabetes, neurodegeneration, and oxidative stress. Although alginate is a high-molecular-weight polymer, its uronic acid residues and oligosaccharide fragments can form hydrogen bonds and ionic interactions with amino acids at enzyme active sites, as previously demonstrated in polysaccharide-protein docking studies [32,33,34]. Therefore, the docking analysis was not intended to predict precise atom-level binding energies for small ligands but rather to support and correlate the experimental bioassay results by visualizing potential interaction sites and mechanisms of inhibition. In our study, alginate displayed moderate binding energies, which were consistent with its measured in vitro activities. The results were therefore used in a comparative and supportive manner to highlight multi-target interactions that justify the observed pharmacological profiles of alginate from *T. ornata* and *H. cuneiformis*, rather than as a stand-alone confirmation of binding strength.

Moreover, molecular docking studies endorsed this, indicating that alginate interacts with key catalytic amino acid residues such as His438 within the BChE active site, although with a higher binding affinity than rivastigmine. On the other hand, TA was markedly more effective in inhibiting COX-1 (IC_50_ = 69.61 µg/mL) and α-amylase (IC_50_ = 45.14 µg/mL), confirming its anti-inflammatory and antidiabetic therapeutic applications. These findings agree with previous studies by Sang et al. [35] and Vo et al. [36], which demonstrated that this inhibitory potential suggests that TA could regulate prostaglandin synthesis pathways and result in reduced inflammation. Also, TA inhibits α-amylase activity, delays carbohydrate digestion, and decreases the spikes of postprandial blood glucose, a mechanism comparable to that of synthetic inhibitors like Acarbose. Interestingly, the binding of alginate to the COX-1 catalytic amino acid residues (GLU 524, PRO 84, ARG 120, and ARG 83, with a docking energy of −6.1609 kcal/mol) and α-amylase (Asp197, HIS 305, and Asp300) mimicked that of aspirin and acarbose, respectively, although at reduced affinity, demonstrating a similar mechanism of inhibition.

The antioxidant activity of the two alginate sample extracts was also assessed. The DPPH scavenging assay is a reliable method of assessing the ability of alginate to act as a free-radical scavenger or hydrogen donor to overcome oxidative stress [37,38,39,40]. The results of the DPPH scavenging assay revealed that TA was again superior to HA, with an IC_50_ of 25.89 µg/mL compared to 58.22 µg/mL, respectively.

These results are consistent with molecular docking analysis showing strong interaction between alginate and antioxidant enzymes such as SOD1 and GPX4. These results agree with the work of Riahi et al. [41] and Bounegru et al. [42], who demonstrated that TA is crucial in preventing oxidative-stress-related diseases such as neurodegenerative disorders, cancer, and cardiovascular disease. Also, the hepatoprotective activity assay showed that TA exhibited a more potent extract (IC_50_ = 118.21 µg/mL) than HA (IC_50_ = 138.36 µg/mL). Although both alginate extracts significantly ameliorated CCl4-induced cytotoxicity in isolated rat hepatocytes, TA’s improved efficacy may result from its ability to decrease oxidative stress, alleviate mitochondrial function, and regulate inflammatory cascades. These results agree with the studies by Atya et al. [43], Tzankova et al. [44] and Bases et al. [38]. It was observed that over increasing alginate concentrations, high viability percentages were detected, which suggests dose-dependent hepatoprotection, confirming alginate’s therapeutic promise. The molecular docking analysis substantiates the in vitro results. Swiss Target Prediction and Gene MANIA analysis clarified alginate’s potential targets, which target diverse protein families, including G-protein-coupled receptors, oxidoreductases, and hydrolases. The key protein targets, including BChE, COX-1, α-amylase, SOD1, and GPX4, were validated via docking analysis. While alginate showed moderate binding energies across targets, consistent hydrogen bonding and ionic interactions were observed, supporting its multi-target therapeutic potential. These results align with Mohammed et al.’s work [45]. Functional enrichment via Gene Ontology recognized involvement in oxidation–reduction processes, inflammatory responses, and metabolic pathway mechanisms relevant to neurodegeneration, diabetes, and hepatic injury.

In conclusion, this research study highlights the therapeutic biochemical relevance of alginates derived from *H. cuneiformis* and *T. ornata*. Comparative analysis shows that although HA displays greater potential in neuroprotection via cholinesterase inhibition, TA displays superior anti-inflammatory, antidiabetic, antioxidant, and hepatoprotective effects. These results underscore the significance of species-specific profiling analysis for brown marine polysaccharide biopolymers and support the strategic selection of alginate sources based on targeted therapeutic biomedical applications.

## 4. Materials and Methods

### 4.1. Collection of Marine Macroalgae

Samples of brown macroalgae, including *Hormophysa cuneiformis* and *Turbinaria ornata*, were collected from the intertidal zone of Hurghada beach, located along the Red Sea coast of Egypt, in January 2024. Firstly, brown macroalgae samples were freshly harvested and were subjected to rinsing with ambient seawater to eliminate debris and epiphytes. Then, these samples were also washed with tap water to remove residual salts and contaminants. The freshly cleaned samples were transported to the laboratory in ice boxes (4 °C) to decrease the metabolic activity and degradation. Taxonomic identification of the algal species was first shown based on morphological characteristics as described in established taxonomic references [46,47,48,49] and followed by further validation using the AlgaeBase online database [49]. Algal samples were dried at room temperature and further dehydrated in a dryer oven at 38 ± 2 °C to confirm sample drying. Then, algal samples were ground, and the seaweed powder was kept in airtight containers to keep their physicochemical characteristics for further bioassays.

### 4.2. Extraction of Polysaccharides from Macroalgae

The sequential extraction protocol is followed to extract alginate, which comprises an initial acidic treatment followed by an alkaline process, as described by Gomaa et al. [50]. Seaweed powder was suspended at a concentration of 1.5% (*w*/*v*) in a 2% (*v*/*v*) citric acid solution and incubated at 30 °C with continuous agitation at 200 rpm for 2 h. During this step, sulfated polysaccharides were released into the extraction solution, while the alginate salts present in the cell wall were converted into insoluble alginic acid. The extraction solution was separated by filtration. After the removal of sulfated polysaccharides, the residual algal biomass was subjected to alkaline treatment (1:40 g/mL) using 2% Na_2_CO_3_ at 40 °C with shaking at 200 rpm for 3 h. Sodium alginate was then precipitated by adding ethanol (1:2, *v*/*v*) and left overnight at 4 °C. The crude sodium alginate was recovered by centrifugation at 6000 rpm for 15 min.

### 4.3. Physicochemical and Structural Characterization

#### 4.3.1. Fourier Transform Infrared Spectroscopy (FTIR) Analysis

FTIR spectroscopy was used to analyze the key functional groups of alginate samples extracted from *H. cuneiformis* and *T. ornata*. The infrared spectra were collected using a Bruker Equinox 55 FTIR spectrophotometer (Bruker Optics GmbH, Ettlingen, Germany) equipped with a high-sensitivity Bauer detector. All samples were scanned within the range of 4000–500 cm^−1^.

#### 4.3.2. X-Ray Diffraction (XRD) Analysis

X-ray diffraction is used to analyze the structural characteristics of two samples of alginate extracted from *Hormophysa cuneiformis* and *Turbinaria ornata*. The alginate powder sample was exposed to structural analysis by using a Panalytical Empyrean 3 X-ray diffractometer (Malvern Panalytical, Almelo, The Netherlands), operating with Cu Kα radiation (λ = 1.542 Å) and equipped with a SolX detector. Scanning was performed over a wide 2θ range to recognize diffraction patterns specific to crystalline or amorphous forms [51].

#### 4.3.3. Thermogravimetric Stability Assessment (TGA)

Both alginate samples were exposed to a Shimadzu TGA-50 thermogravimetric analyzer (Shimadzu Corporation, Kyoto, Japan) to analyze their thermal behavior.

Approximately 5 mg of each dried sample was heated in a nitrogen atmosphere (10 mL/min) from 50 °C up to 800 °C [52]. The observed mass loss data support the insights into the decomposition stages and the whole thermal resilience of each alginate sample.

#### 4.3.4. Elemental Composition Analysis

Alginate samples were subjected to CHNS elemental analyzers to evaluate the basic chemical constituents inside alginate samples [53]. Alginate samples were dried at 60 °C in an oven to remove moisture and then eventually milled. Alginate samples were burned in the analyzer, and the gases produced were separated, measured, and quantified to find out how much carbon (C), hydrogen (H), nitrogen (N), and sulfur (S) were present by weight.

#### 4.3.5. Monosaccharide Profiling via HPLC

High-Performance Liquid Chromatography (HPLC) was conducted to explore the sugar monomers composing alginate polymers extracted from *H. cuneiformis* and *T. ornata*. To conduct analysis, both an Agilent 1100 system (Agilent, Santa Clara, CA, USA) outfitted with dual pumps and a UV-visible detector were used. Separation of sugar components is facilitated using a reversed-phase C18 column (4.6 mm × 260 mm, 5 µm). HPLC is conducted by using 2 mobile phases. The first mobile phase is solvent A, which consists of acetonitrile. The second phase is solvent B, which consists of a mixture of purified water and acetonitrile (90:10 *v*/*v*) with buffering agents KH_2_PO_4_ and triethylamine attuned to pH 7.5. A gradient elution was applied across 20 min at a flow rate of 1.0 mL/min, and detection occurred at 245 nm [54].

#### 4.3.6. Quantification of Uronic Acid Content

The HPLC method is used to determine uronic acid concentrations in both alginate samples extracted from *Hormophysa cuneiformis* and *Turbinaria ornata*. The chromatographic technique is carried out by using a C18 column (250 mm × 4.6 mm), and the applicable mobile phase is 0.01 M sulfuric acid at a steady flow rate of 0.5 mL/min. 25 µL of the prepared alginate samples was injected manually through each run [5]. Detection wavelengths were improved to suit uronic acid peaks, enabling precise quantification of this important functional group.

### 4.4. Computational Analysis

Computational docking analysis is used to predict the interaction between alginate and several proteins including crystal structure of human butyryl cholinesterase in complex with a choline molecule (PDB: 1P0M), structure of Human Pancreatic alpha-Amylase in complex with the carbohydrate Inhibitor Acarbose (PDB: 1b2y), human COX-1 crystal structure (PDB: 6Y3C), crystal structure of human Superoxide Dismutase I (hSOD1) in complex with a naphthalene-catechol linked compound (PDB: 5YTO), and crystal structure of human GPX4 in complex with GXpep-1 (PDB: 5H5Q). The alginate ligand (G-M dimer) was modeled using software such as Molecular Operating Environment (MOE) and Discovery Studio Visualizer, following traditional protein preparation (water removal and addition of hydrogen atoms). Docking was performed using the Molecular Operating Environment software (MOE, version 2015.10) and BIOVIA Discovery Studio Visualizer, version 2020. All structure minimizations were performed until an RMSD gradient of 0.05 kcal∙mol^−1^ Å^−1^ with the MMFF94x force field, and partial charges were automatically calculated. All intervening water molecules were removed from the structure, and then the target protein was prepared for docking using the Protonate 3D protocol in MOE with default parameters. The co-crystalized ligand was used to define the binding site for the docking simulation. The Triangle Matcher Placement method and London dG scoring function were employed for docking and scoring. The docking protocol was first validated by self-docking the co-crystallized ligand near the protein’s binding site [55]. The ligand-receptor interactions at the protein binding site were studied with the validated docking protocol (RMSD < 2) for the reported inhibitors to predict their binding mode and binding affinity.

### 4.5. Butyl Choline Esterase Inhibitory Activity Assay

Alginate extract stock was first prepared (10 mg/mL) by solubilizing in dimethyl sulfoxide (DMSO). After stock preparation, the stocks are diluted serially by using Tris-HCl buffer (pH 8.0) to obtain the final test concentration range (0.25–500 μg/mL). To avoid the effect of DMSO, the assay mixtures were formulated so that the final DMSO concentration did not exceed 1% (*v*/*v*) in any well. The enzyme inhibition assay was evaluated using equine butyrylcholinesterase (BChE; Sigma) and the spectrophotometric Ellman’s method [12,56]. BChE inhibition assay was performed in 48-well microplates. Each well has a 0.22 U/mL equine BChE in Tris-HCl buffer (pH 8.0). The assay was conducted by adding test Alginate extract dilutions or control solutions to the wells, and the enzyme-inhibitor mixtures were incubated at 37 °C for 20 min. The reaction of the BChE inhibition assay was initiated by adding substrate (0.5 mM butyryl thiocholine iodide) and 0.35 mM 5,5′-dithiobis-(2-nitrobenzoic acid) (DTNB, Ellman’s reagent; Sigma). The production of thiocholine was regulated by recording the increase in absorbance at 412 nm on a BioTek TS 800 microplate reader (BioTek, Winooski, VT, USA) over 15 min. Positive control, Rivastigmine (Sigma), was included in wells of the assay from a range (0.25–500 μg/mL), and negative control wells did not contain the inhibitor. All samples and controls were performed in triplicate. The results of the percentage inhibition at each concentration were calculated relative to the negative control. Half-maximal inhibitory concentration (IC_50_) values were calculated by plotting dose–response curves and fitting the data through GraphPad Prism (version 6, GraphPad Software). IC_50_ values represent the mean ± standard deviation from three independent experiments (n = 3).

### 4.6. Anti-Inflammatory Activity

Algal alginate samples were assessed for their inflammatory effects in vitro by analyzing their interaction with the cyclooxygenase-1 (COX-1) enzyme using a screening assay kit (catalog no. K548; Biovision, Milpitas, CA, USA) following the manufacturer’s protocol [57]. Alginate samples were dissolved in 1.0% (*v*/*v*) dimethyl sulfoxide (DMSO) at a range from 0.25 to 500 μg/mL, making the final volume adjusted to 1 mL. A positive control sample, diclofenac, was included, and negative control wells do not contain inhibitors. GraphPad Prism software was used to calculate the half-maximal inhibitory concentration (IC_50_) values from the dose–response curves [57].

### 4.7. Antioxidant Assay

Algal alginate samples were assessed for their antioxidant potential by using the 2,2-diphenyl-1-picrylhydrazyl (DPPH) free radical scavenging method at the Regional Center for Mycology and Biotechnology (RCMB), Al-Azhar University. All samples were performed in triplicate, and the data were expressed as mean values. Firstly, the DPPH solution is freshly prepared at a concentration of 0.004% (*w*/*v*) methanolic solution at 10 °C in the dark until use. Then, Alginate samples were prepared in a methanolic solution at the preferred concentration. Aliquot of methanolic mixture (40 μL) was added to 3 mL of the DPPH solution. The reaction mixture was immediately exposed to spectrophotometric investigation using a UV-visible spectrophotometer (Milton Roy, Spectronic 1201, Milton Roy, Houston, TX, USA), with the decrease in absorbance monitored at 515 nm. Optical density or absorbance readings were recorded at 1-min intervals for up to 16 min, or until stabilization of the signal. Positive control, ascorbic acid, was also included [58,59] by adding DPPH solution without the addition of any antioxidant. The percentage of radical scavenging activity was calculated using the following equation:

(1) $$PI\;\left(\%\right)=(\;\frac{Ac-AT}{Ac}\;)\;\;\times \;100$$

where *Ac* is the absorbance of the control at time zero, and AT is the absorbance of the test sample at 16 min. IC_50_, or the half-maximal inhibitory concentration, is the concentration of extract needed to achieve 50% inhibition of DPPH radicals, which was determined from dose–response curves using GraphPad Prism software (GraphPad Software, San Diego, CA, USA).

### 4.8. Antidiabetic Activity

The colorimetric method using 3,5-dinitrosalicylic acid (DNSA) is used to analyze the inhibitory effect of alginate samples towards α-amylase activity. Alginate samples were prepared by dissolving in a minimal volume of 10% dimethyl sulfoxide (DMSO), followed by dilution with phosphate buffer (Na_2_HPO_4_/NaH_2_PO_4_, 0.02 M; NaCl, 0.006 M; pH 6.9) to achieve final concentrations ranging from 10 to 1000 μg/mL. For each assay, 200 μL of the alginate sample was mixed with 200 μL of α-amylase solution (2 U/mL) and incubated at 30 °C for 10 min. Then, 200 μL of a 1% starch solution was added to the reaction mixture to initiate the enzymatic reaction, which was incubated for 3 min. The enzymatic reaction was stopped by adding 200 μL of DNSA reagent and heating in a water bath at 85–90 °C for 10 min. DNSA reagent was prepared by dissolving 12 g of sodium potassium tartrate tetrahydrate in 8.0 mL of 2 M NaOH and 20 mL of 96 mM DNSA solution. After heating, alginate samples were allowed to cool at room temperature, the reaction mixture was diluted with 5 mL of distilled water, and the absorbance was measured at 540 nm. A positive control sample (Acarbose) was included, and it contained all the contents of the reaction mixture without the alginate samples [60,61]. Negative control or blank does not contain the enzyme to account for background absorbance. The percentage of α-amylase inhibition was calculated using the following formula:

(2) $$Inhibition\;\left(\%\right)=(\;\frac{Ac-AT}{Ac}\;)\;\times \;100$$

where *Ac* is the absorbance of the control (enzyme activity without inhibitor), and AT is the absorbance in the presence of the test sample. Dose–response curves were produced by plotting the percentage inhibition against the logarithm of sample concentrations. The IC_50_ values are defined as the concentration required to inhibit 50% of enzyme activity, which were derived from the fitted curves using GraphPad Prism software.

### 4.9. Hepatoprotective Study in Hepatocytes Using MTT Assay

#### 4.9.1. Isolation of Rat Hepatocytes

The collagenase perfusion method was used to isolate hepatocytes from male Wistar rats [62] according to ethical approval no. IACUC-SCI-TU-0492. The isolated hepatocyte cells were resuspended in Krebs–Henseleit buffer (pH 7.4), supplemented with 12.5 mM HEPES (Sigma-Aldrich, Gillingham, UK), reaching a final density of 1 × 10^6^ cells/mL. Cell suspensions were preserved at 37 °C in special conditions, including a humidified incubator (95% O_2_ and 5% CO_2_), to confirm physiological conditions.

#### 4.9.2. Experimental Design

Isolated hepatocyte cultures were subjected to a medium containing 1% (*v*/*v*) carbon tetrachloride (CCl_4_), either alone or in combination with various concentrations (2, 10, 50, 100, 250, 500, and 1000 µg/mL) of the test alginate samples.

The experimental design included the following treatment groups:

Group 1: Untreated control hepatocytes

Group 2: Hepatocytes treated with 1% CCl_4_ (negative control)

Group 3: Hepatocytes treated with 1% CCl_4_ and the reference hepatoprotective agent, silymarin

Group 4: Hepatocytes treated with 1% CCl_4_ and *T. ornata* alginate extract

Group 5: Hepatocytes treated with 1% CCl_4_ and *H. cuneiformis* alginate extract

Each experimental condition was performed in quadruplicate (three wells per treatment per replicate) to confirm accuracy, reproducibility, and statistical reliability.

#### 4.9.3. MTT Viability Assay

The enzymatic activity of mitochondrial dehydrogenase is a scale to quantify cell viability using MTT [62,63]. Hepatocytes were seeded by adding 100 μL of hepatocyte suspension (1 × 10^6^ cells/mL) to each well. After hepatocytes were treated with alginate extracts, 50 μL of MTT solution (5 mg/mL in PBS; Sigma-Aldrich) was added to each well. Treated Plates were gently shaken and incubated in the dark at 37 °C for 4 h in a 5% CO_2_ atmosphere. Then, after incubation, violet formazan crystals were observed inside the wells. 150 μL of DMSO (Sigma-Aldrich) was added to solubilize crystals, and the absorbance was measured at 570 nm using a microplate reader (SunRise, Tecan, Morrisville, NC, USA). Formation of formazan or reduction in MTT by metabolically active cells offers a quantitative measure of cell viability, mitochondrial integrity, and overall cytoprotective response.

Cell viability (%) was calculated using the formula:

(3) $$Cell\;viability\;\left(\%\right)=(\;\frac{OD\;test}{OD\;control}\;)\times \;100$$

where OD test is the mean optical density of wells treated with the test compound, and OD control is the mean optical density of untreated control cells.

The hepatoprotective effect was expressed as the difference in viability between treated and CCl_4_-only groups, calculated as:

Hepatoprotective Effect (%) = Viability treatment − Viability CCl_4_ group

The 50% effective concentration (EC_50_), defined as the concentration at which 50% restoration of cell viability relative to control cells was observed, was calculated from dose–response curves using GraphPad Prism software (GraphPad Software, San Diego, CA, USA). Results were expressed as mean ± standard deviation.

### 4.10. Statistical Analysis

Unless specified, all experimental data were obtained from a minimum of 3–5 replicates per concentration. Data are presented as mean ± SD. Statistical differences among groups were assessed using one-way ANOVA followed by Tukey’s post hoc test for multiple comparisons (GraphPad Software, 10.4.1. San Diego, CA, USA). A *p*-value < 0.05 was considered statistically significant. Different letters above data points or bars indicate significant differences according to Tukey’s test.

## 5. Conclusions

This study provides a comparative evaluation of alginate extracted from *T. ornata* and *H. cuneiformis*, highlighting species-specific physicochemical characteristics and therapeutic profiles. Physicochemical analyses (FTIR, XRD, TGA, elemental profiling, HPLC) revealed differences in uronic acid content, monosaccharide composition, crystallinity, and thermal stability that correlated with biological functions. *T. ornata* alginate exhibited stronger antioxidant, anti-inflammatory, antidiabetic, and hepatoprotective activities, while *H. cuneiformis* alginate showed superior neuroprotective potential via butyrylcholinesterase inhibition. Molecular docking and network analyses supported these results, confirming interactions with key therapeutic targets (COX-1, BChE, α-amylase, SOD1, GPX4) and involvement in oxidative stress, inflammation, and metabolic regulation. Overall, alginates emerge as multi-target marine polysaccharides with distinct, source-dependent bioactivities. These findings emphasize the importance of species selection in alginate-based drug development and warrant further in vivo validation and formulation studies to explore clinical applications.

## Figures and Tables

**Table 1:** Thermal decomposition stages and weight loss characteristics of alginate extracted from *Hormophysa cuneiformis* and *Turbinaria ornata* samples.

Samples	1st Stage (°C)	Weight Loss 1 (%)	2nd Stage (°C)	Weight Loss 2 (%)	3rd Stage (°C)	Weight Loss 3 (%)	Total Weight Loss (%)
HA	45–167	8.95	168–581	52.41	582–996	29.57	90.93
TA	46–338	22.92	338–654	20.42	655–989	47.95	90.79

**Table 2:** Elemental composition of alginate extracted from *H. cuneiformis* (HA) and *T. ornata* (TA).

Sample	C%	H%	N%	S%
HA	11.98	2.16	2.69	0.97
TA	11.19	2.29	5.19	1.63

**Table 3:** Quantitative Analysis of Monosaccharides and Uronic Acid of alginate extracts from *H. cuneiformis* (HA) and *T. ornata* (TA) determined by HPLC (concentrations in µg/g).

Sugar	HA (µg/g)	TA (µg/g)
Rhamnose	4.78	3.55
Galactose	7.74	6.37
Fucose	3.23	4.71
Fructose	4.02	-
Glucose	11.32	2.84
Mannose	4.39	3.01
Uronic acid	10.69	11.85

**Table 4:** Cellular targets of Alginate predicted by Swiss prediction.

Alginate Target Proteins	Common Name	Uniport ID	ChEMBL ID	Target Class	Probability
Alginate					
P-glycoprotein 1	ABCB1	P08183	CHEMBL4302	Primary active transporter	0.11573667
Gamma-secretase	PSEN2 PSENEN NCSTN APH1A PSEN1 APH1B	P49810 Q9NZ42 Q92542 Q96BI3 P49768 Q8WW43	CHEMBL2094135	Protease	0.11573667
Protein kinase C alpha	PRKCA	P17252	CHEMBL299	Kinase	0.11573667
Kappa Opioid receptor	OPRK1	P41145	CHEMBL237	Family A G protein-coupled receptor	0.11573667
Protein kinase C delta (by homology)	PRKCD	Q05655	CHEMBL2996	Kinase	0.11573667
Cytochrome P450 19A1	CYP19A1	P11511	CHEMBL1978	Cytochrome P450	0.11573667
Serotonin 2b (5-HT2b) receptor	HTR2B	P41595	CHEMBL1833	Family A G protein-coupled receptor	0.11573667
Alpha-2a adrenergic receptor	ADRA2A	P08913	CHEMBL1867	Family A G protein-coupled receptor	0.11573667
Adrenergic receptor alpha-2	ADRA2C	P18825	CHEMBL1916	Family A G protein-coupled receptor	0.11573667
Alpha-2b adrenergic receptor	ADRA2B	P18089	CHEMBL1942	Family A G protein-coupled receptor	0.11573667
Dopamine D1 receptor	DRD1	P21728	CHEMBL2056	Family A G protein-coupled receptor	0.11573667
Dopamine D2 receptor	DRD2	P14416	CHEMBL217	Family A G protein-coupled receptor	0.11573667
Alpha-1d adrenergic receptor	ADRA1D	P25100	CHEMBL223	Family A G protein-coupled receptor	0.11573667
Serotonin 2a (5-HT2a) receptor	HTR2A	P28223	CHEMBL224	Family A G protein-coupled receptor	0.11573667
Serotonin 2c (5-HT2c) receptor	HTR2C	P28335	CHEMBL225	Family A G protein-coupled receptor	0.11573667
Dopamine D3 receptor	DRD3	P35462	CHEMBL234	Family A G protein-coupled receptor	0.11573667
Cytochrome P450 2D6	CYP2D6	P10635	CHEMBL289	Cytochrome P450	0.11573667
Serotonin 6 (5-HT6) receptor	HTR6	P50406	CHEMBL3371	Family A G protein-coupled receptor	0.11573667
Alpha-1a adrenergic receptor (by homology)	ADRA1A	P35348	CHEMBL229	Family A G protein-coupled receptor	0.11573667
Serotonin 1b (5-HT1b) receptor (by homology)	HTR1B	P28222	CHEMBL1898	Family A G protein-coupled receptor	0.11573667
Transient receptor potential cation channel subfamily V member 4 (by homology)	TRPV4	Q9HBA0	CHEMBL3119	Voltage-gated ion channel	0.11573667
Protein phosphatase 2C alpha	PPM1A	P35813	CHEMBL2437	Phosphatase	0.11573667
Glucose transporter	SLC2A1	P11166	CHEMBL2535	Electrochemical transporter	0.11573667
Brain adenylate cyclase 1	ADCY1	Q08828	CHEMBL2899	Enzyme	0.11573667
Protein phosphatase 2C beta	PPM1B	O75688	CHEMBL2845	Phosphatase	0.11573667
Protein-tyrosine phosphatase 1B	PTPN1	P18031	CHEMBL335	Phosphatase	0.11573667
Serine/threonine protein phosphatase PP1-gamma catalytic subunit	PPP1CC	P36873	CHEMBL4438	Phosphatase	0.11573667

**Table 5:** Docking energy scores and amino acids are involved in the binding site for alginate with the active site of the target protein.

Crystal Structure of Human Butyryl Cholinesterase in Complex with a Choline Molecule (PDB: 1P0M)				
Compound/ Drug	Docking Score (kcal/mol)	Amino Acids Involved in Binding	2D	3D
**Rivastigmine** **(Control)**	−6.6908 kcal/mol	GLY ^117^ (A) H-acceptor	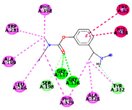	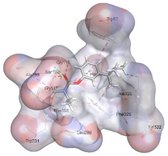
**Alginate**	−7.5459 kcal/mol	HIS ^438^ (A) ionic	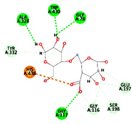	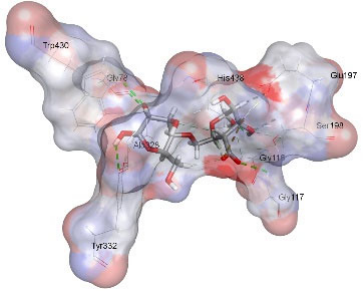
**Structure of human pancreatic alpha-amylase in complex with the carbohydrate inhibitor acarbose** **(PDB: 1b2y)**				
**Co-crystalized ligand**	−8.5414 kcal/mol	GLU ^233^ (A) H-donor LYS ^200^ (A) H-acceptor GLN ^63^ (A) H-acceptor TRP ^59^ (A) H-pi TRP ^59^ (A) H-pi	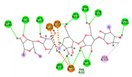	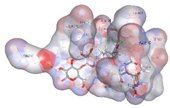
**Acarbose** **(Control)**	−8.8135 kcal/mol	HIS ^201^ (A) H-donor GLU ^233^ (A) H-donor GLU ^233^ (A) H-donor ASP ^197^ (A) H-donor ASP ^300^ (A) H-donor ASP ^197^ (A) H-donor TRP ^59^ (A) H-donor THR ^163^ (A) H-donor LYS ^200^ (A) H-acceptor ARG ^195^ (A) H-acceptor HIS ^299^ (A) H-acceptor HIS ^305^ (A) H-acceptor GLN ^63^ (A) H-acceptor GLU ^233^ (A) ionic GLU ^233^ (A) ionic ASP ^300^ (A) ionic TYR ^62^ (A) H-pi	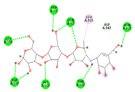	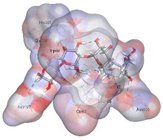
**Alginate**	−6.5440 kcal/mol	ASP ^197^ (A) H-donor ASP ^197^ (A) H-donor ASP ^300^ (A) H-donor HIS ^305^ (A) H-acceptor HIS ^305^ (A) ionic HIS ^305^ (A) ionic	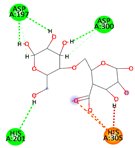	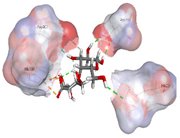
**Human COX-1 Crystal Structure (PDB: 6Y3C)**				
**Co-crystalized ligand**	−4.1243 kcal/mol	ARG ^83^ (A) H-acceptor ARG ^83^ (A) H-acceptor ARG ^83^ (A) ionic ARG ^83^ (A) ionic ARG ^120^ (A) ionic ARG ^120^ (A) ionic	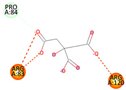	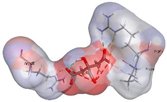
**Aspirin** **(Control)**	−4.8185 kcal/mol	ARG ^120^ (A) ionic	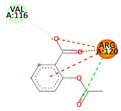	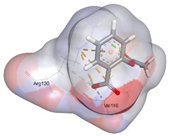
**Alginate**	−6.1609 kcal/mol	GLU ^524^ (A) H-donor PRO ^84^ (A) H-donor ARG ^120^ (A) H-acceptor ARG ^83^ (A) H-acceptor ARG ^83^ (A) H-acceptor ARG ^120^ (A) ionic ARG ^120^ (A) ionic ARG ^120^ (A) ionic	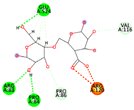	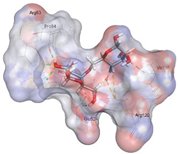
**Crystal Structure of human Superoxide Dismutase I (hSOD1) in complex with a napthalene-catechol linked compound (PDB: 5YTO)**				
**Co-crystalized ligand**	−3.6198 kcal/mol	GLN ^15^ (D) H-donor GLY ^10^ (D) H-donor	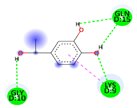	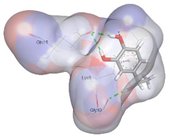
**Vitamin C** **(control)**	−3.9119 kcal/mol	ASP ^11^ (D) H-donor GLY ^12^ (D) H-donor GLY ^10^ (D) H-acceptor	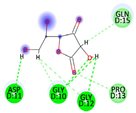	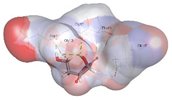
**Alginate**	−5.0492 kcal/mol	GLY ^10^ (D) H-donor ASN ^53^ (D) H-acceptor LYS ^9^ (D) ionic	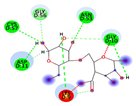	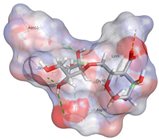
**Crystal structure of human GPX4 in complex with GXpep-1 (PDB: 5H5Q)**				
**Vitamin C** **(control)**	−3.7975 kcal/mol	ASP ^128^ (A) H-donor ASP ^128^ (A) H-donor LYS ^58^ (A) H-acceptor	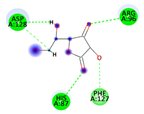	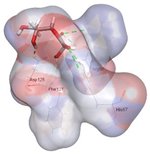
**Alginate**	−4.7917 kcal/mol	HIS ^42^ (A) H-donor ARG ^60^ (A) H-acceptor ARG ^60^ (A) ionic	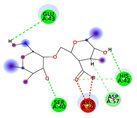	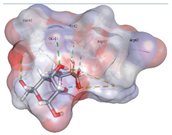

**Table 6:** Comparative Potency between Alginate Extracted from *Hormophysa cuneiformis* and *Turbinaria ornata*.

Activity	*H. cuneiformis* Alginate (IC_50_ µg/mL)	*T. ornata* Alginate (IC_50_ µg/mL)	More Potent
BChE Inhibition	39.01	107.38	HA
COX-1 Inhibition	360.22	69.61	TA
α-Amylase Inhibition	341.48	45.14	TA
Antioxidant Activity	58.22	25.89	TA
Hepatoprotective Activity	138.36	118.21	TA

## Data Availability

The original contributions presented in this study are included in the article. Further inquiries can be directed to the corresponding author.

## Abbreviations

The following abbreviations are used in this manuscript:

Abbreviation	Definition
BChE	Butyrylcholinesterase
COX-1	Cyclooxygenase-1
DPPH	2,2-Diphenyl-1-picrylhydrazyl
DNSA	3,5-Dinitrosalicylic acid
FTIR	Fourier Transform Infrared Spectroscopy
XRD	X-ray Diffraction
HPLC	High-Performance Liquid Chromatography
DTNB	5,5-Dithiobis-(2-nitrobenzoic acid)
MTT	3-(4,5-Dimethylthiazol-2-yl)-2,5-Diphenyltetrazolium Bromide
ROS	Reactive Oxygen Species
MOE	Molecular Operating Environment
GPX4	Glutathione Peroxidase 4
SOD1	Superoxide Dismutase 1
T. Alginate	Alginate from *Turbinaria ornata*
TGA	Thermogravimetric Analysis
Tris-HCl	Tris(hydroxymethyl)aminomethane Hydrochloride Buffer
UV	Ultraviolet
IC_50_	Half Maximal Inhibitory Concentration
EC_50_	Half Maximal Effective Concentration
PDB	Protein Data Bank
Akt	Protein Kinase B
Nrf2	Nuclear Factor Erythroid 2-Related Factor 2
NF-κB	Nuclear Factor Kappa B
PI3K	Phosphoinositide 3-Kinase
HA	*Hormophysa cuneiformis* alginate
TA	*Turbinaria ornata* alginate
