# Correction: El-Sheekh et al. Therapeutic Evaluation of Alginate from Brown Seaweeds: A Comparative Study of *Turbinaria ornata* and *Hormophysa cuneiformis*. *Pharmaceuticals* 2025, *18*, 1720

**DOI:** 10.3390/ph19091396

**Published:** 2026-09-03

**Authors:** Mostafa M. El-Sheekh, Eman Bases, Shimaa M. El Shafay, Rania A. El-Shenody, Mostafa E. Elshobary, Abdel Hady A. Abdel Wahab, Wesam E. Yousuf, Dorya I. Essa, Samar Sami Alkafaas

**Affiliations:** 1 Botany and Microbiology Department, Faculty of Science, Tanta University, Tanta 31527, Egypt; mostafaelsheikh@science.tanta.edu.eg (M.M.E.-S.); eman.bases@science.tanta.edu.eg (E.B.); shimaa.elshafay@science.tanta.edu.eg (S.M.E.S.); rania.abdelsamad@science.tanta.edu.eg (R.A.E.-S.); dorya_eesa@science.tanta.edu.eg (D.I.E.); 2 Aquaculture Research, Alfred Wegener Institute (AWI)–Helmholtz Centre for Polar and Marine Research, Am Handelshafen, 27570 Bremerhaven, Germany; 3 Department of Cancer Biology, National Cancer Institute, Cairo University, Cairo 11796, Egypt; abdelhady.abdelwahab@nci.cu.edu.eg; 4 Biochemistry Division, Chemistry Department, Faculty of Science, Tanta University, Tanta 31527, Egypt; wessam-pg147370@scienc.tanta.edu.eg; 5 Molecular Cell Biology Unit, Division of Biochemistry, Department of Chemistry, Faculty of Science, Tanta University, Tanta 31527, Egypt; samar.alkafas@science.tanta.edu.eg

**Figure Legend**

In the original publication [1], there was an error in the legends of Figures 7–11. The y-axis was incorrectly labeled as “conc (mg/mL)”; the correct label should read “Inhibition (%)”. The correct legend appears below. 

**Figure 7:**
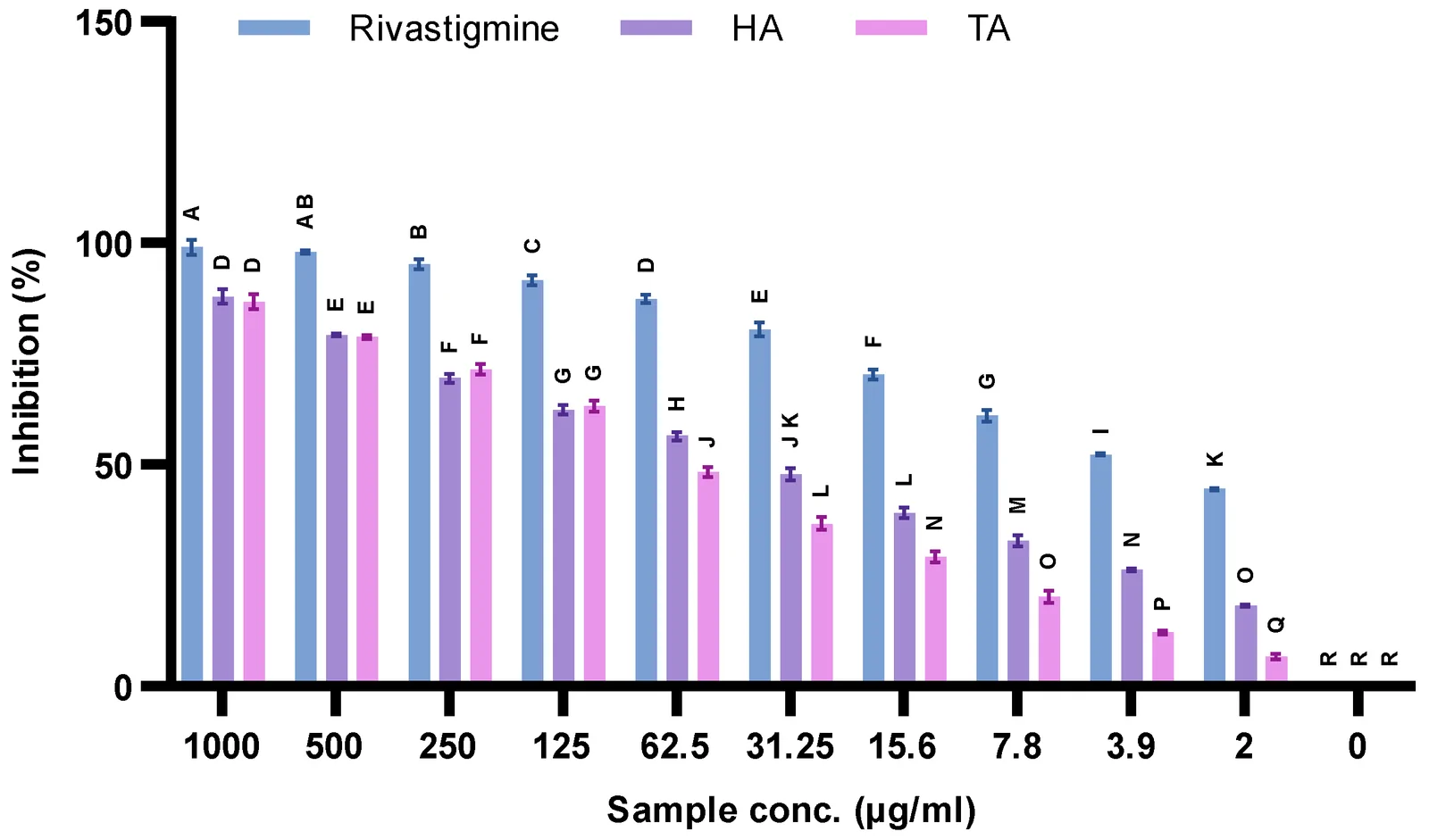
Inhibition of butyryl choline esterase (BChE) by alginate extracted from *Hormophysa cuneiformis* and *Turbinaria ornata* compared with rivastigmine. Data are presented as mean ± SD (n = 3). Different letters above the bars indicate statistically significant differences among groups according to one-way ANOVA followed by Tukey’s post hoc test (*p* < 0.05).

**Figure 8:**
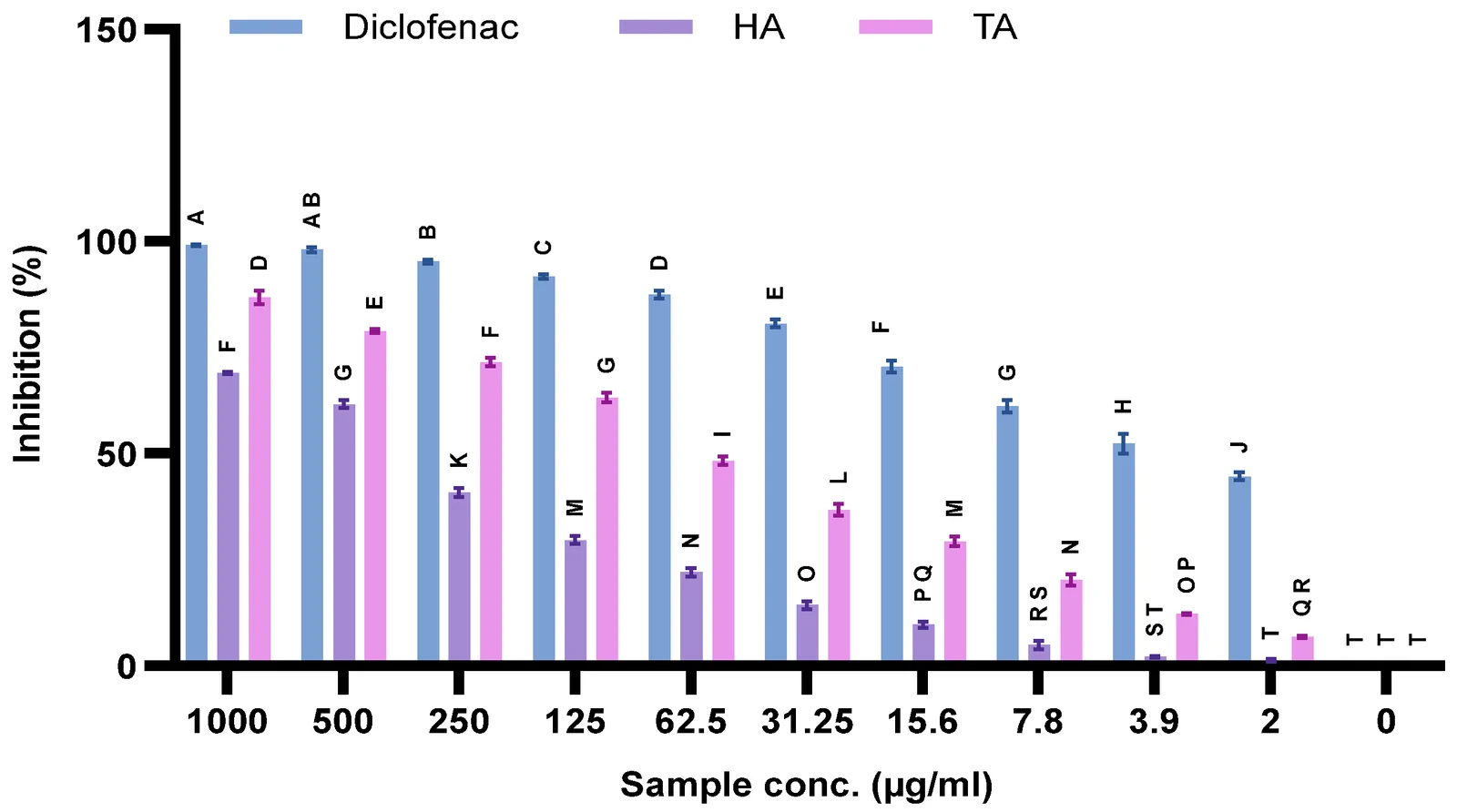
Inhibition of COX-1 activity by alginate extracted from *H. cuneiformis* (HA) and *Turbinaria ornata* (TA) compared with Diclofenac. Data are presented as mean ± SD (n = 3). Different letters above the bars indicate statistically significant differences among groups according to one-way ANOVA followed by Tukey’s post hoc test (*p* < 0.05).

**Figure 9:**
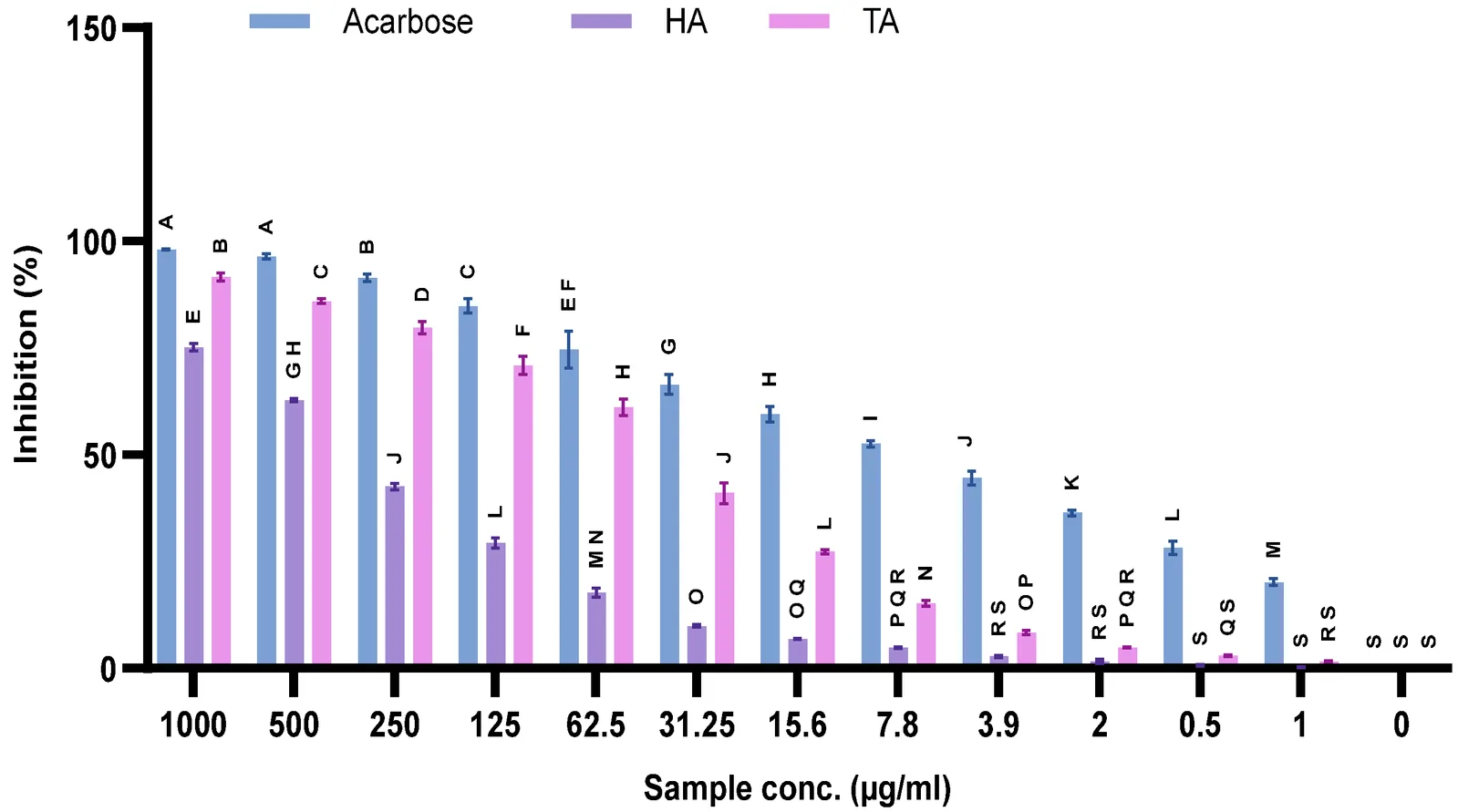
Inhibition of α-amylase activity by alginate extracted from *H. cuneiformis* (HA) and *T. onata* (TA) compared with Acarbose. Data are presented as mean ± SD (n = 3). Different letters above the bars indicate statistically significant differences among groups according to one-way ANOVA followed by Tukey’s post hoc test (*p* < 0.05).

**Figure 10:**
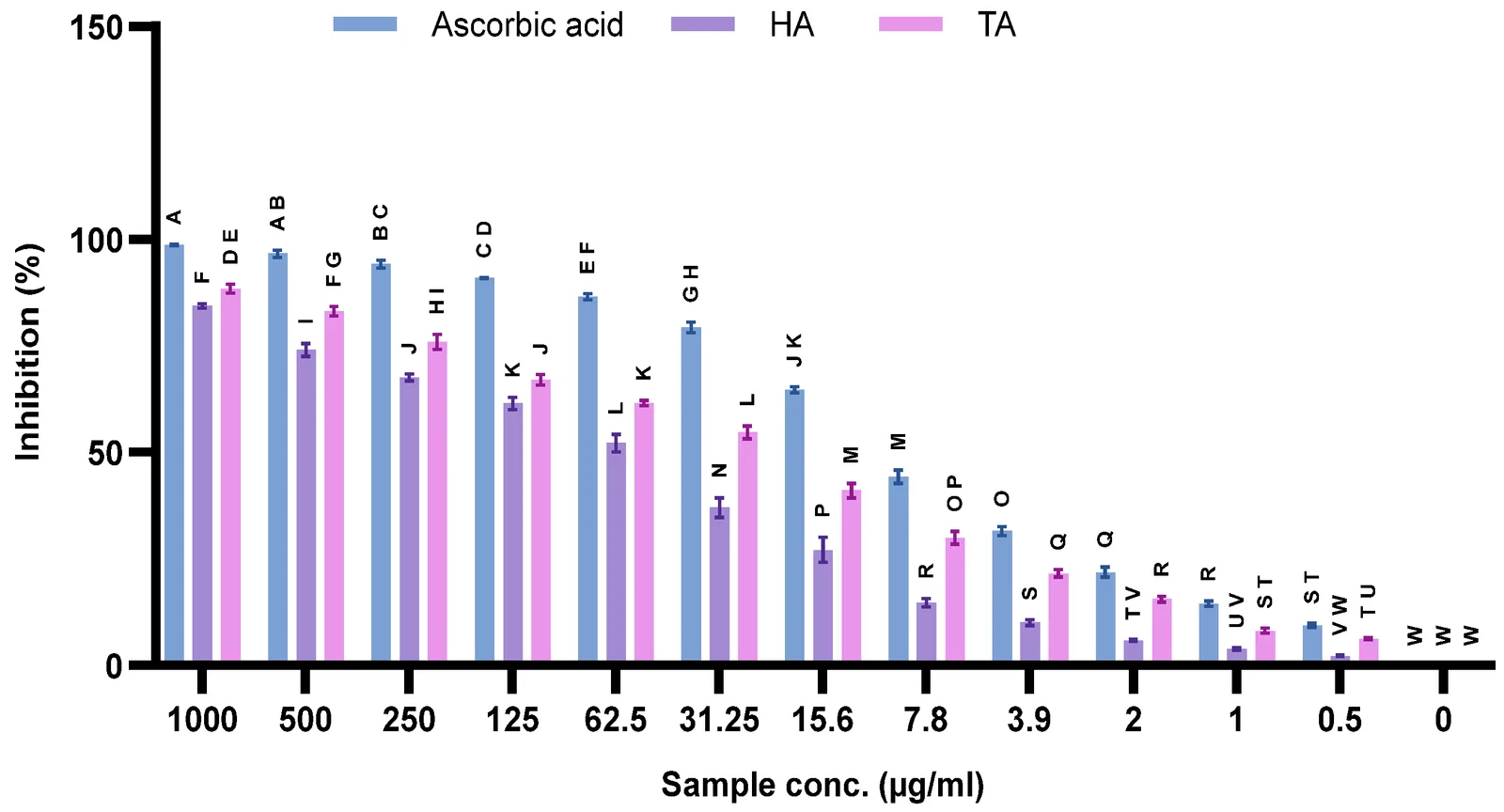
Inhibition of DPPH activity by alginate extracted from *H. cuneiformis* (HA) and *T. onata* (TA) compared with Ascorbic acid standard. Data are presented as mean ± SD (n = 3). Different letters above the bars indicate statistically significant differences among groups according to one-way ANOVA followed by Tukey’s post hoc test (*p* < 0.05).

**Figure 11:**
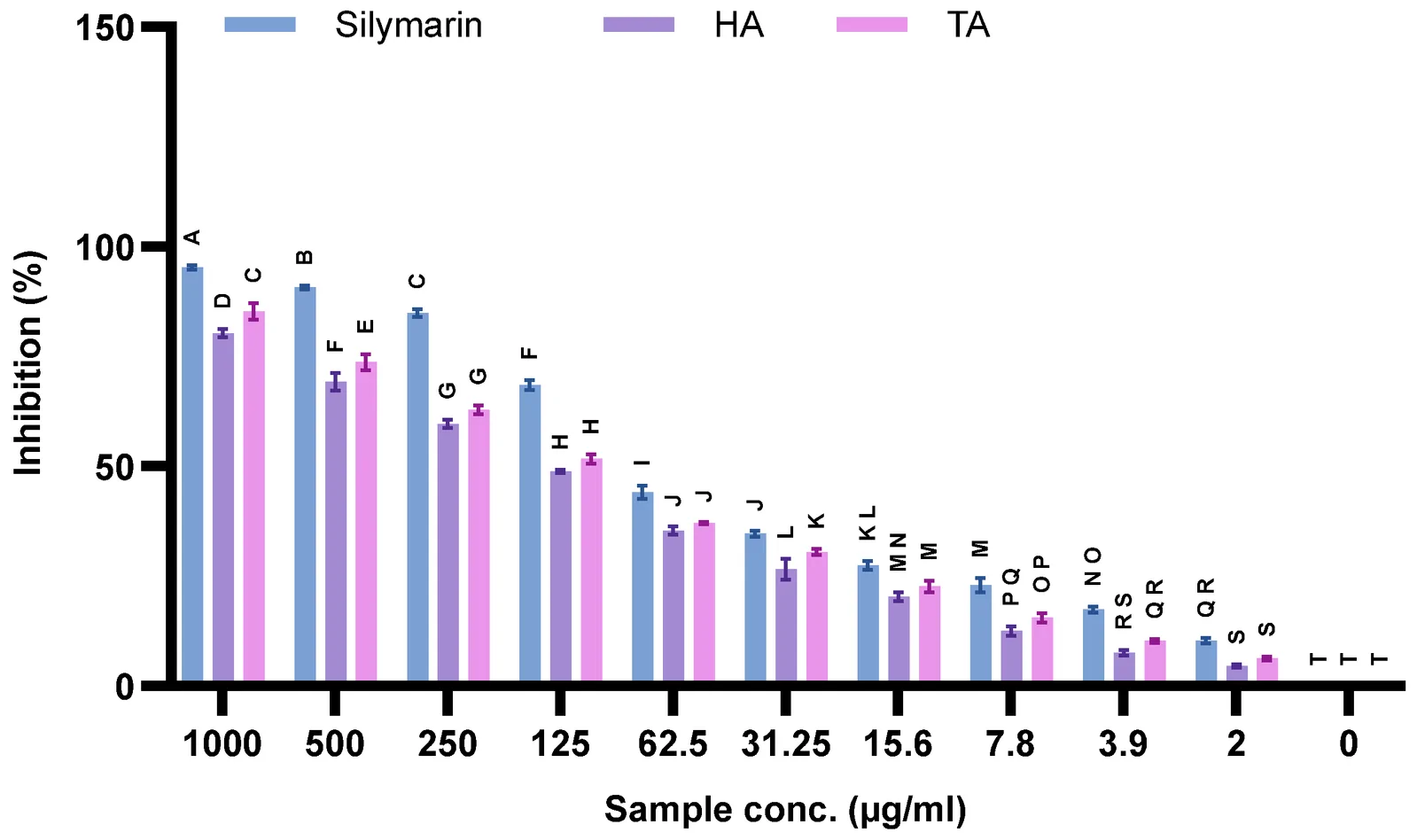
Inhibition of α-amylase activity by alginate extracted from *H. cuneiformis* (HA) and *T. onata* (TA) compared with Silymarin. Data are presented as mean ± SD (n = 3). Different letters above the bars indicate statistically significant differences among groups according to one-way ANOVA followed by Tukey’s post hoc test (*p* < 0.05).

**Text Correction**

There was an error in the original publication [1]. The statements referring to sulfur content and the presence of sulfated polysaccharides in alginate were scientifically inaccurate, as alginate is a non-sulfated polysaccharide. Any sulfur signal, if detected, can be attributed to trace residues of proteins or amino acids rather than the alginate structure itself. To avoid potential scientific misinterpretation, these sentences have been removed.

A correction has been made to Section 3 (Discussion), Paragraphs 9–11:

Moreover, molecular docking studies endorsed this, indicating that alginate interacts with key catalytic amino acid residues such as His438 within the BChE active site, although with a higher binding affinity than rivastigmine. On the other hand, TA was markedly more effective in inhibiting COX-1 (IC_50_ = 69.61 µg/mL) and α-amylase (IC_50_ = 45.14 µg/mL), confirming its anti-inflammatory and antidiabetic therapeutic applications. These findings agree with previous studies by Sang et al. [35] and Vo et al. [36], which demonstrated that this inhibitory potential suggests that TA could regulate prostaglandin synthesis pathways and result in reduced inflammation. Also, TA inhibits α-amylase activity, delays carbohydrate digestion, and decreases the spikes of postprandial blood glucose, a mechanism comparable to that of synthetic inhibitors like Acarbose. Interestingly, the binding of alginate to the COX-1 catalytic amino acid residues (GLU 524, PRO 84, ARG 120, and ARG 83, with a docking energy of −6.1609 kcal/mol) and α-amylase (Asp197, HIS 305, and Asp300) mimicked that of aspirin and acarbose, respectively, although at reduced affinity, demonstrating a similar mechanism of inhibition.

The antioxidant activity of the two alginate sample extracts was also assessed. The DPPH scavenging assay is a reliable method of assessing the ability of alginate to act as a free-radical scavenger or hydrogen donor to overcome oxidative stress [37–40]. The results of the DPPH scavenging assay revealed that TA was again superior to HA, with an IC_50_ of 25.89 µg/mL compared to 58.22 µg/mL, respectively.

These results are consistent with molecular docking analysis showing strong interaction between alginate and antioxidant enzymes such as SOD1 and GPX4. These results agree with the work of Riahi et al. [41] and Bounegru et al. [42], who demonstrated that TA is crucial in preventing oxidative-stress-related diseases such as neurodegenerative disorders, cancer, and cardiovascular disease. Also, the hepatoprotective activity assay showed that TA exhibited a more potent extract (IC_50_ = 118.21 µg/mL) than HA (IC_50_ = 138.36 µg/mL). Although both alginate extracts significantly ameliorated CCl4-induced cytotoxicity in isolated rat hepatocytes, TA’s improved efficacy may result from its ability to decrease oxidative stress, alleviate mitochondrial function, and regulate inflammatory cascades. These results agree with the studies by Atya et al. [43], Tzankova et al. [44] and Bases et al. [38]. It was observed that over increasing alginate concentrations, high viability percentages were detected, which suggests dose-dependent hepatoprotection, confirming alginate’s therapeutic promise. The molecular docking analysis substantiates the in vitro results. Swiss Target Prediction and Gene MANIA analysis clarified alginate’s potential targets, which target diverse protein families, including G-protein-coupled receptors, oxidoreductases, and hydrolases. The key protein targets, including BChE, COX-1, α-amylase, SOD1, and GPX4, were validated via docking analysis. While alginate showed moderate binding energies across targets, consistent hydrogen bonding and ionic interactions were observed, supporting its multi-target therapeutic potential. These results align with Mohammed et al.’s work [45]. Functional enrichment via Gene Ontology recognized involvement in oxidation–reduction processes, inflammatory responses, and metabolic pathway mechanisms relevant to neurodegeneration, diabetes, and hepatic injury.

The authors state that the scientific conclusions are unaffected. This correction was approved by the Academic Editor. The original publication has also been updated.
